# Lighting-Up Tumor for Assisting Resection via Spraying NIR Fluorescent Probe of γ-Glutamyltranspeptidas

**DOI:** 10.3389/fchem.2018.00485

**Published:** 2018-10-12

**Authors:** Haidong Li, Qichao Yao, Feng Xu, Ning Xu, Wen Sun, Saran Long, Jianjun Du, Jiangli Fan, Jingyun Wang, Xiaojun Peng

**Affiliations:** ^1^State Key Laboratory of Fine Chemicals, Dalian University of Technology, Dalian, China; ^2^Department School of Life Science and Biotechnology, Dalian University of Technology, Dalian, China

**Keywords:** enzyme-activated, NIR fluorescent probe, spraying, large stokes shift, diagnose, clinical practice, tumor, γ-Glutamyltranspeptidase

## Abstract

For the precision resection, development of near-infrared (NIR) fluorescent probe based on specificity identification tumor-associated enzyme for lighting-up the tumor area, is urgent in the field of diagnosis and treatment. Overexpression of γ-glutamyltranspeptidase, one of the cell-membrane enzymes, known as a biomarker is concerned with the growth and progression of ovarian, liver, colon and breast cancer compared to normal tissue. In this work, a remarkable enzyme-activated NIR fluorescent probe NIR-SN-GGT was proposed and synthesized including two moieties: a NIR dicyanoisophorone core as signal reporter unit; γ-glutamyl group as the specificity identification site. In the presence of γ-GGT, probe NIR-SN-GGT was transformed into NIR-SN-NH_2_, the recovery of Intramolecular Charge Transfer (ICT), liberating the NIR fluorescence signal, which was firstly employed to distinguish tumor tissue and normal tissues *via* simple “spraying” manner, greatly promoting the possibility of precise excision. Furthermore, combined with magnetic resonance imaging by T2 weight mode, tumor transplanted BABL/c mice could be also lit up for first time by NIR fluorescence probe having a large stokes, which demonstrated that probe NIR-SN-GGT would be a useful tool for assisting surgeon to diagnose and remove tumor in clinical practice.

## Introduction

With the improvement of economic level, diagnose and treatment of cancer is getting more and more attention (Cheng et al., 2011; Chen et al., 2016, 2018; Cong et al., 2018; Jung et al., 2018; Lee et al., 2018; Yang et al., 2018; Zhang et al., 2018). The traditional clinical detection techniques, such as positron emission tomography (PET) (Mileshkin et al., 2011; Xiao et al., 2012), magnetic resonance imaging (MRI) (Sardanelli et al., 2010; Pinto et al., 2011) and X-ray imaging (Huang et al., 2011; Shi et al., 2014), are widely used to the medical examination of humans. Unfortunately, the fatal flaw in these techniques is the inability to diagnose early tumors, especially for flat or depressed lesions (Park et al., 2018). Although histopathological biopsy could provide the accurate diagnose result, it is suffered from the possibility of real-time diagnosis *in situ* due to the complex processing and time-consuming of the sample separation (Kozlowski et al., 2006). Indeed, surgical specialists urgent need an effective tool to easily distinguish between tumor and normal tissue during surgery. Fluorescence technology has been paid unprecedented attention (Blum et al., 2007; Paulick and Bogyo, 2008; Asanuma et al., 2015; Ofori et al., 2015; Cheng et al., 2016; Gu et al., 2016a,b; Hu et al., 2016; Chen et al., 2017; He et al., 2017; Liu et al., 2017a,b,c; Wang et al., 2017a, 2018; Yin et al., 2017; Kuriki et al., 2018; Li et al., 2018b; Llancalahuen et al., 2018; Miao et al., 2018; Sedgwick et al., 2018; Shang et al., 2018; Verwilst et al., 2018), especially for medical diagnosis and treatment system (Kim et al., 2017; Li et al., 2017b; Verwilst et al., 2017), owing to efficient identification without destructive *in vivo*. Thus, it is of great medical significance to develop fluorescent probe that can assist clinician for imaging and resection of tumor.

γ-glutamyltranspeptidase (γ-GGT; EC 2.3.2.2), an over-expression enzyme associated with the growth and progression of ovarian, liver, colon and breast cancer compared to normal tissue (De Young et al., 1978; Shinozuka et al., 1979; Rao et al., 1986), plays important roles in many physiological and pathological processes (Pompella et al., 2006; Strasak et al., 2010), which is regarded as an important target for analysis and imaging *in vitro* or *in vivo* (Li et al., 2015b, 2016c; Wang et al., 2015, 2017b; Park et al., 2016a,b; Zhang et al., 2016a,b; Liu et al., 2018). However, rarely related fluorescent probe could be qualified for the identification tumor by tracking the activity of γ-GGT enzyme *in vivo* (Urano et al., 2011), especially for NIR emission property, possessing good performances of avoiding auto-fluorescence and deeper tissue penetration (Gu et al., 2016a). In addition, large stokes shift could effectively avoid self-absorbing for beneficial to fluorescence imaging. Till to now, there is no fluorescence probe visualized tracking the activity of γ-GGT enzyme in various organs. More importantly, by “spraying” manner, tumor tissue could be conveniently and efficiently lighted up from normal tissue, which is urgent need for precision medicine.

In this work, based on our previous research (Li et al., 2018a), through installing γ-GGT enzyme-activable unit on photo-stability; large stokes shift and controllable NIR chromophore, probe NIR-SN-GGT was successfully proposed and synthesized for monitoring the activity of γ-GGT *in vitro* and *in vivo*. NIR fluorescence emission was strictly modulated through the electron donor capability of amino. Upon addition γ-GGT into solution, obvious NIR fluorescence emission signal was observed after activable group cut off, which attributed to the enhancement of electronic capacity of product NIR-SN-NH_2_. By layer scanning and 3D imaging construction of tissue slices, the probe NIR-SN-GGT was breakthrough employed to evaluate the content of γ-GGT enzyme in different organs. To the best of our knowledge, tumor tissue could be distinguished from normal through “spraying” NIR fluorescence probe style for the first time.

### Experimental section

#### General information and materials

All reagents used were obtained from commercial suppliers and were used without further purification unless otherwise stated. Solvents used were purified *via* standard methods. Twice-distilled purified water used in all experiments was from Milli-Q systems. ^1^H-NMR and ^13^C-NMR spectra were recorded on a Bruker Avance II 400 MHz spectrometer. Chemical shifts (δ) were reported as ppm (in MeOD or DMSO-*d*_6_, with TMS as the internal standard). Fluorescence spectra were performed on a VARIAN CARY Eclipse fluorescence spectrophotometer (Serial No.MY15210003) in 10 × 10 mm quartz cell. Excitation and emission slit widths were modified to adjust the fluorescence intensity to a suitable range. Absorption spectra were measured on Agilent Technologies CARY 60 UV-Vis spectrophotometer (Serial No.MY1523004) in 10 × 10 mm quartz cell. Mass spectrometric data were achieved with HP1100LC/MSD MS and an LC/Q-TOF-MS instruments. Mito-Tracker Green, Lyso-Tracker Green and Hoechst 33342 were purchased from Life Technologies Co. (USA). Nitroreductase, transglutaminase, γ-glutamyltranspeptidase, and 6-Diazo-5-oxo-L-norleucine (DON) were purchased from Sigma-Aldrich. All pH measurements were performed using the Ohaus Starter 2100 pH meter. The fluorescence quantum yields for compounds with Absolute PL Quantum Yield Spectrometer (HAMAMATSU C11347). Instruments used in cell imaging tests were carried out on Olympus FV1000 and FV1000-IX81 confocal microscopy (Olympus, Japan). Slight pH variations in the solutions were achieved by adding the minimum volumes of HCl or NaOH (1 M). Flash column chromatography was performed using silica gel (200–300 mesh) obtained from Qingdao Ocean Chemicals. Flow cytometry analysis was carried out on cytometer (Attune NxT). MRI imaging of mice was carried out on NIUMAG analytical instrument (MesoMR23-060H-I). Tumor tissue slices were prepared from freezing microtome (LEICA CM1860 UV). All the interferential reagents were prepared based on published literatures (Li et al., 2015a, 2016a,b, 2018a; Fan et al., 2017).

#### Detection limit

The detection limit (DL) was calculated based on the fluorescence titration of probe NIR-SN-GGT (10 μM) in the presence of γ-GGT (0-10 mU/mL). The fluorescence intensity of probe NIR-SN-GGT was measured and standard deviation of the blank measurement was achieved. The detection limit was calculated with the following equation: Detection limit = 3σ/*k*. Where σ is the standard deviation of the blank measurement, *k* is the slope between the fluorescence intensity (F_650_ nm) versus various γ-GGT concentrations.

#### Determination of the quantum yield

The fluorescence quantum yields for compounds with Absolute PL Quantum Yield Spectrometer (HAMAMATSU C11347). Operating this system is simple. Load a sample and press the start button to measure the photoluminescence quantum yields, excitation wavelength dependence, PL excitation spectrum and other properties in a short time. The PL Quantum Yield (Φ) is expressed as the ratio of the number of photons emitted from molecules (PNem) to that absorbed by molecules (PNabs). As following equation: Φ = PNem/PNabs.

#### Cell incubation

Ovarian cancer cells (A2780 cells), breast cancer 4T1 cells (4T1 cells) and human umbilical vein endothelial cells (HUVEC cells) were purchased from Institute of Basic Medical Sciences (IBMS) of the Chinese Academy of Medical Sciences. Except for A2780 cells treated with Dulbecco's modified Eagle's medium (DMEM, Invitrogen), others cells were cultured in RPMI medium 1640 supplemented with 10% fetal bovine serum (Invitrogen). The cells were seeded in confocal culture dishes and then incubated for 24 h at 37°C under a humidified atmosphere containing 5% CO_2_.

#### Cytotoxicity assays

Measurement of cell viability was measured by reducing of MTT (3-(4, 5)-dimethylthiahiazo (-2-yl)-3, 5-diphenytetrazoliumromide) to formazan crystals using mitochondrial dehydrogenases. A2780 and HUVEC cells were seeded in 96-well microplates (Nunc, Denmark) at a density of 1 × 10^5^ cells/mL in 100 μL medium containing 10% FBS. After 24 h of cell attachment, the plates were then washed with 100 μL/well PBS buffer. The cells were then cultured in medium with 0, 1, 2, 5, and 10 μM of probe NIR-SN-GGT for 24 h. Cells in culture medium without probe NIR-SN-GGT were used as the control. Six replicate wells were used for each control and test concentration. 10 μL of MTT (5 mg/mL) prepared in PBS was added to each well and the plates were incubated at 37°C for another 4 h in a 5% CO_2_ humidified incubator. The medium was then carefully removed, and the purple crystals were lysed in 200 μL DMSO. Optical density of solutions was determined on a microplate reader (Thermo Fisher Scientific) at 490 nm. Cell viability was expressed as a percent of the control culture value, and it was calculated using the following equation: Cells viability (%) = (OD_dye_ – OD_blank_)/ (OD_control_ – OD_blank_) × 100

#### Imaging endogenous γ-GGT activity in living cells

A2780 and HUVEC cells were seeded in glass-bottom culture dishes at approximately concentration of 2 × 10^4^ cells/mL and allowed to culture for 24 h at 37°C in a 5% CO_2_ humidified incubator. For the detection of endogenous γ-GGT activity, A2780 and HUVEC cells were incubated with probe NIR-SN-GGT (10 μM) 37°C for 30 min, followed by washing thrice with free DMEM. Under the confocal fluorescence microscope (Olympus FV1000-IX81) with a 60 × objective lens, probe NIR-SN-GGT was excited at 488 nm (one-photon) and 800 (two-photon), next, fluorescence emission at 655–755 nm channel and 575–630 nm channel were gathered, respectively.

#### Time-dependent cell imaging

The time-dependent cell imaging of probe NIR-SN-GGT (10 μM) monitoring endogenous γ-GGT activity in A2780 cells and HUVEC cells were investigated on Olympus FV1000-IX81 with the excitation at 488 nm. Fluorescent signals were recorded as time went on. After that, quantitative image analysis of the average fluorescence intensity of cells, determined from analysis of 9 regions of interest (ROIs) across cells.

#### Concentration-dependent cell imaging

The concentration-dependent cell imaging of probe NIR-SN-GGT for monitoring endogenous γ-GGT activity in A2780 cells was investigated on Olympus FV1000-IX81 with the excitation at 488 nm. In the experiment, the concentration of probe NIR-SN-GGT was set 0, 5, 10, and 15 μM, respectively. Before cell imaging, A2780 cells were pre-treated with probe NIR-SN-GGT for 30 min at 37°C in the incubator. After that, quantitative image analysis of the average fluorescence intensity of cells, determined from analysis of 9 regions of interest (ROIs) across cells.

#### Flow cytometry analysis

A2780 cells and HUVEC cells gathered in logarithmic growth phase were incubated in 6-well with DMEM medium or RPMI 1640 medium at 37°C in a 5% CO_2_ humidified incubator. The density of 1 × 10^5^-5 × 10^5^ was cultured and dilution with 500 μL PBS buffer. A2780 cells and HUVEC cells were pre-treated with probe NIR-SN-GGT (10 μM) 37°C for 30 min, followed by centrifugation (1000 r/min, 5 min) and dispersion with PBS buffer solution for twice. After that, cells were finally suspended in 0.5 ml PBS buffer and analyzed through flow cytometer (Attune NxT), and each sample was terminated with 10,000 target cells.

#### Visualization of γ-GGT activity in tissue and mice xenograft tumor model

All procedures were carried out in compliance with the guide for the care and use of laboratory animal resources and the national research council, and were approved by the institutional animal care and use committee of the NIH. For establishing a mouse tumor model, the 4T1 mammary carcinoma cells were chose to transplant under the armpit of approximately 15–20 g male BABL/c mice. After 10 days inoculation, the xenograft tumor mice were given with 50 μM 100 μL probe NIR-SN-GGT through *in-situ* injection within the period of mice anesthesia. After that, the imaging of mice was carried out on the NightOWL II LB983 small animal *in vivo* imaging system (Germany) with a 475 nm excitation and a 665 nm emission filter. Tumor tissue slices of 100 or 500 μm were prepared from freezing microtome (LEICA CM1860 UV). As a comparison, normal slices were chose from muscle tissue of hind legs. Next, these tissues were incubated with probe NIR-SN-GGT (10 μM) at 37°C for 45 min, followed by washing thrice with phosphate buffer saline (0.01 M, pH 7.4). Under the confocal fluorescence microscope (Olympus FV1000-IX81) with a 60 × objective lens, probe TCF-GGT was excited at 800 (two-photon), next, fluorescence emissions at 575–630 nm of red channel were gathered. In tissue depth imaging, the 3D images were constructed *via* every 5 μm as a step under the same condition. Quantitative image analysis of the average fluorescence intensity of cells, determined from analysis of 9 regions of interest (ROIs) across cells.

#### Synthesis of probe NIR-SN-GGT

##### Synthesis of compound 2

Compound **1** (2.76 g, 20 mmol), malononitrile (3.30 g, 50 mmol) and catalytic dose of piperidine (0.05 mL) were dissolved in 50 mL dry ethanol. The mixture was stirred at 80°C for 6 h. After removing the solvent by reduced pressure distillation, fuscous solid was obtained finally. The solid was with ethyl acetate three times. The organic phase was dried with anhydrous Na_2_SO_4_ for overnight and vacuum filter. Following, the crude product was purified through silica gel column chromatography to obtain 1.93 g white crystal compound **2** (yield 52%). ^1^H NMR (400 MHz, DMSO-*d*_6_) δ 6.56 (d, *J* = 1.3 Hz, 1H), 2.54 (s, 2H), 2.24 (s, 2H), 2.05 (s, 3H), 0.96 (s, 6H). ^13^C NMR (100 MHz, DMSO-*d*_6_) δ 171.91, 162.98, 119.87, 113.92, 113.12, 76.48, 45.28, 42.43, 32.42, 27.74, 25.43, ESI-MS: *m*/*z* calculated for C_12_H_13_${\text{N}}_{2}^{-}$ [M-H]^−^: 185.25, found: 185.12.

##### Synthesis of compound 5 and 6

The synthesis of compound **5** and **6** were referred from the reported literature (Li et al., 2018a).

##### Synthesis of compound Boc-NIR-SN-GGT

Compound **6** (406 mg, 1 mmol) and compound **2** (186 mg, 1 mmol) were dissolved in 5 mL dry EtOH solution. Then, catalytic dose of piperidine (0.05 mL) was added into the above solution. The reaction system was stirred at 80°C for overnight under N_2_ protection. After removing the solvent by reduced pressure distillation, crude jacinth solid was obtained finally without further purification for the next step. ESI-MS: *m*/*z* calculated for C_33_H_42_N_4_${\text{NaO}}_{5}^{+}$ [M+Na]^+^: 597.30, found: 597.32.

##### Synthesis of probe NIR-SN-GGT

Compound **Boc-NIR-SN-GGT** (56 mg, 0.1 mmol) was dissolved in 2 mL dry CH_2_Cl_2_ and stirred at room temperature for 10 min. Next, 2 mL CH_2_Cl_2_-TFA (v/v 1:1) was added into above mixture *via* drop by drop style. When add was completed, the mixture system continued to stir overnight. After that, the crude product was purified through silica gel column chromatography to obtain 23 mg bright red probe **NIR-SN-GGT** (yield 54%)0.1H NMR (400 MHz, MeOD/TFA, 0.60 mL/0.05 mL) δ 7.62 (d, *J* = 8.7 Hz, 2H), 7.56 (d, *J* = 8.8 Hz, 2H), 7.17 (d, *J* = 16.1 Hz, 1H), 7.07 (d, *J* = 16.1 Hz, 1H), 6.81 (s, 1H), 4.07 (t, *J* = 6.5 Hz, 1H), 2.71 (t, *J* = 7.0 Hz, 2H), 2.60 (s, 2H), 2.53 (s, 2H), 2.27 (m, 2H), 1.06 (s, 6H). ^13^C NMR (125 MHz, MeOD-TFA) δ 171.19, 170.11, 169.85, 155.21, 139.67, 136.70, 131.90, 128.13, 122.47, 119.77, 116.62, 113.78, 112.46, 76.98, 52.07, 42.52, 38.45, 31.47, 26.63, 25.48. ESI-HRMS: *m*/*z* calculated for C_24_H_27_N_4_${\text{O}}_{3}^{+}$ [M+H]^+^: 419.2078, found: 419.2074.

## Results and discussion

### Design probe NIR-SN-GGT

**Scheme 1:**
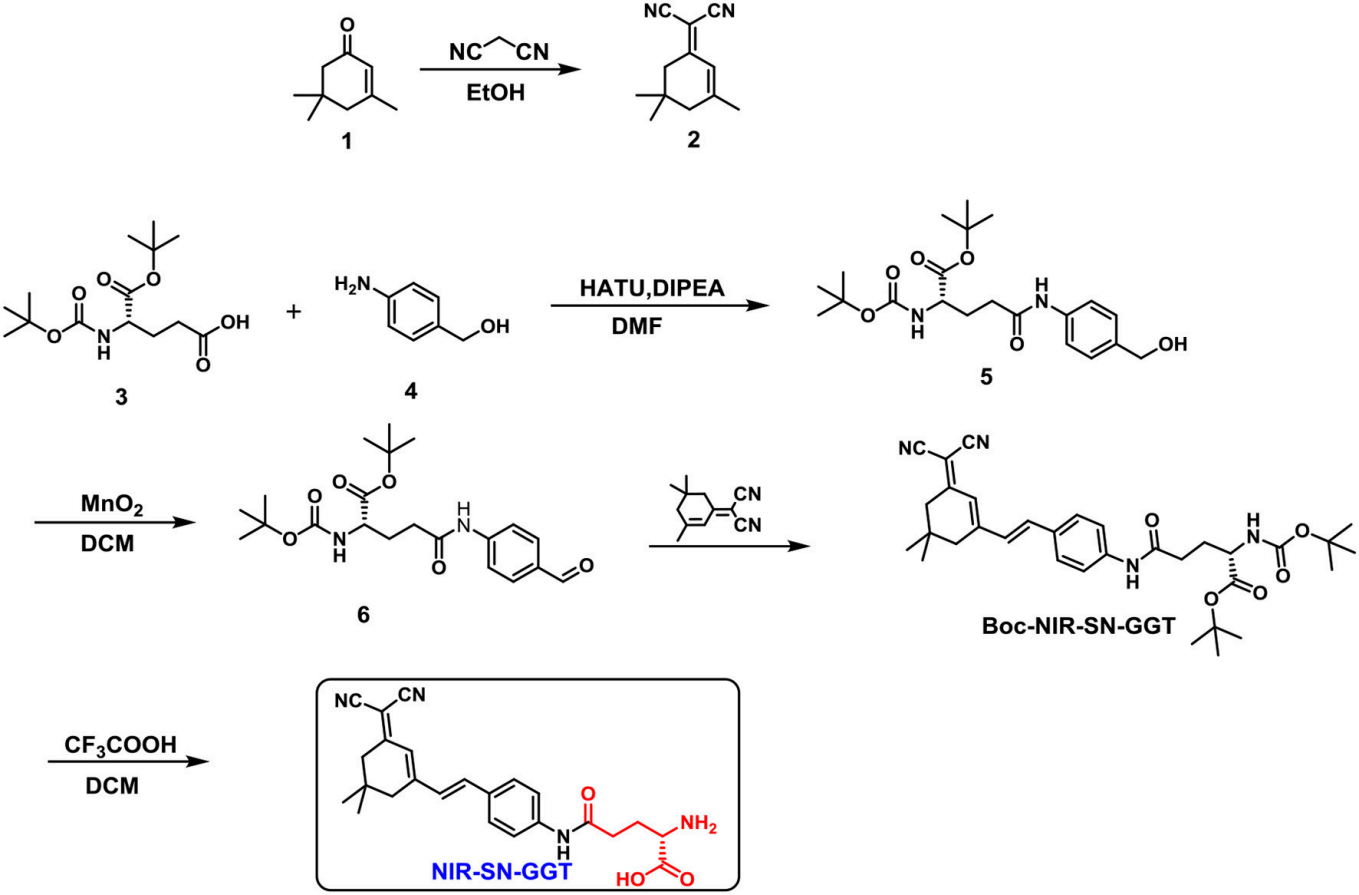
The synthesis route of probe NIR-SN-GGT.

**Scheme 2:**
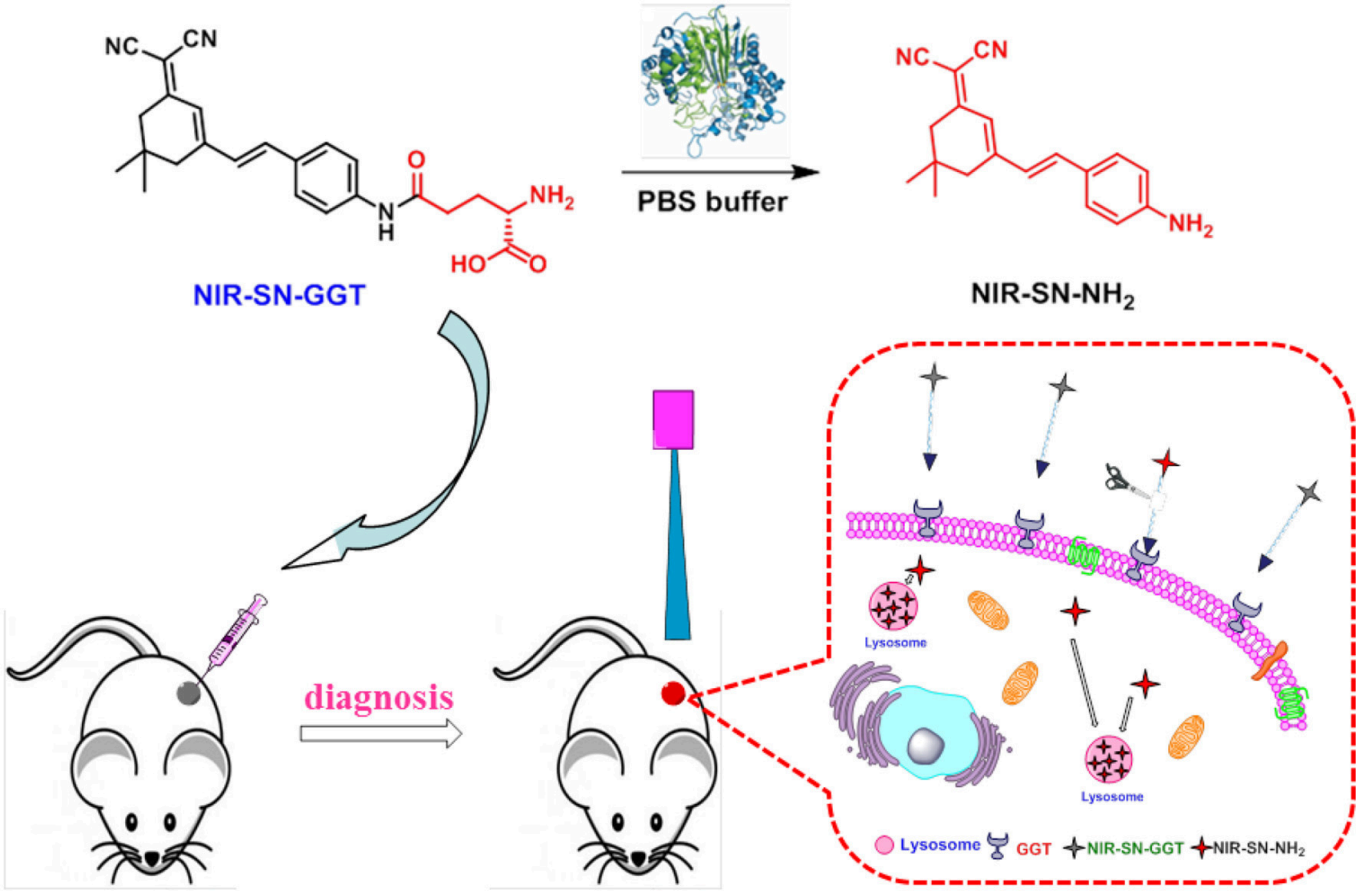
Recognition mechanism of probe NIR-SN-GGT for γ-GGT enzyme *in vivo*.

The synthetic procedure of probe NIR-SN-GGT was described in Scheme 1, which was fully validated through ^1^H, ^13^C-NMR and ESI-HRMS in the Supplementary Data (Figures S20–S22). In consideration of γ-glutamyl group as the recognition, it was installed on the framework of near-infrared emission. When γ-GGT encounter probe NIR-SN-GGT, it is cut off by specificity, liberating bare amino in the D-π-A structure. Then, NIR fluorescence signal of around 650 nm was observed through the modulation of Intermolecular Charge Transfer (ICT), where the recognition procedure of probe NIR-SN-GGT for target was shown in Scheme 2.

### Spectroscopic characteristics of probe NIR-SN-GGT

**Figure 1:**
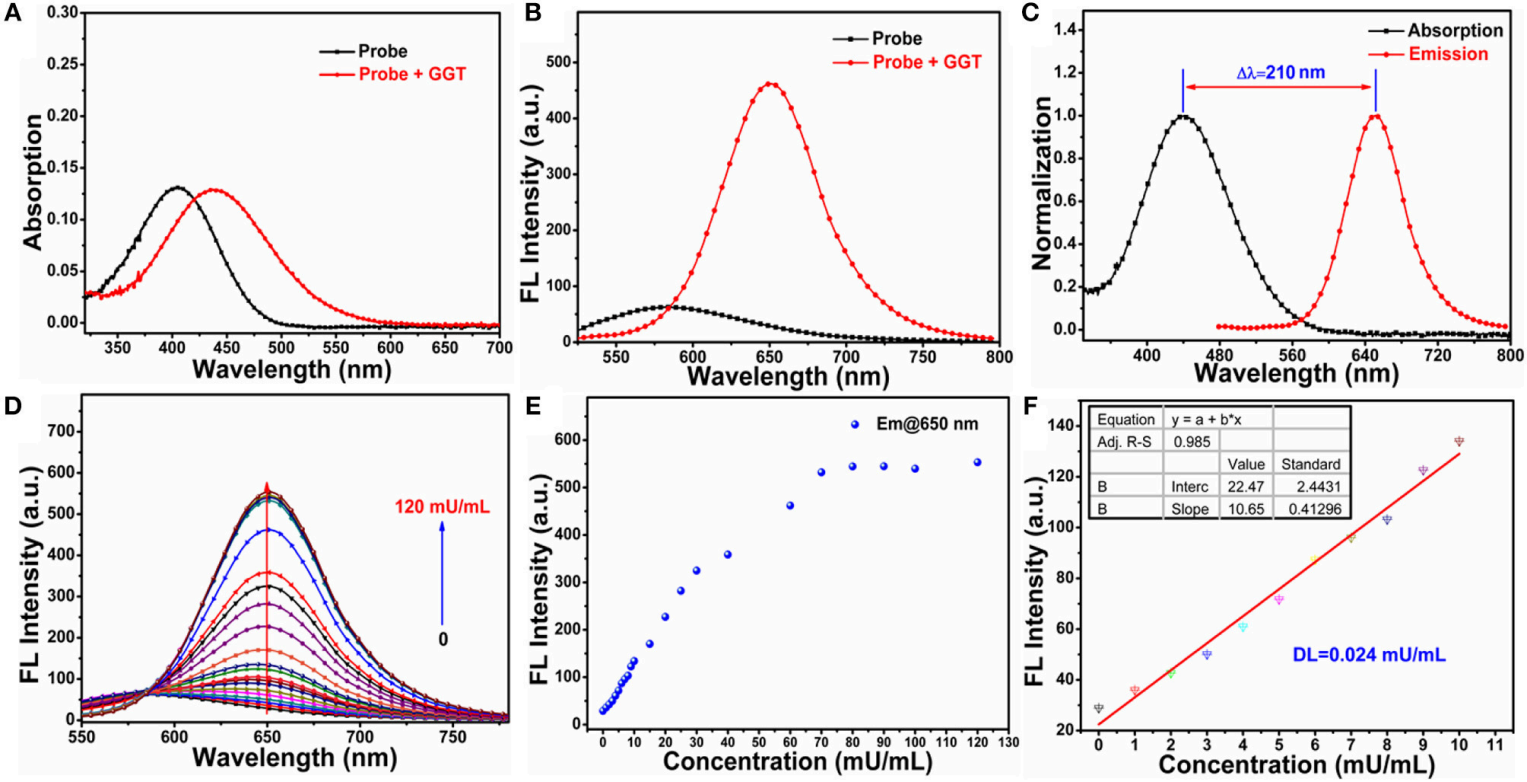
**(A)** UV-Vis and **(B)** fluorescence spectra changes of probe NIR-SN-GGT (10 μM) toward adding 60 mU/mL γ-GGT enzyme in PBS buffer solution (0.01 M, pH 7.4). **(C)** Normalization UV-Vis (blank line) and fluorescence spectrum (red line) of NIR-SN-NH_2_ in PBS buffer solution (0.01 M, pH 7.4). **(D)** Fluorescence titration experiments of probe NIR-SN-GGT (10 μM) toward different concentrations of γ-GGT enzyme (titration concentrations: 0, 1, 2, 3, 4, 5, 6, 7, 8, 9, 10, 15, 20, 25, 30, 40, 60, 70, 80, 90, 100, 120 mU/mL). **(E)** Fluorescence intensities of probe NIR-SN-GGT (10 μM) around 650 nm toward various concentrations of γ-GGT enzyme (0-120 mU/mL). **(F)** The linearity of F_650nm_ vs. low concentration of γ-GGT ranging from 0 to 10 mU/mL. The experiments were repeated three times and the data were shown as mean (±S.D.). λ_ex_ = 445 nm, slit: 10/10 nm.

With probe NIR-SN-GGT in hand, firstly, we studied the basic spectral properties of probe NIR-SN-GGT in different solvents (Figures S1, S2). UV-Vis titration experiments of probe NIR-SN-GGT in PBS buffer solution (0.01 M, pH 7.4) demonstrated that probe had considerable solubility in aqueous solution (Figure S3). In addition, probe NIR-SN-GGT showed good stability (Figure S4), which laid the foundation for practical application of biology. As is seen in Figure 1A, adding 60 mU/mL γ-GGT enzyme in PBS buffer solution, and the absorption peak around 405 nm of probe NIR-SN-GGT (10 μM) reduced accompanied by the appearance of new absorption peak around 440 nm. The color of solution ranging from light green to yellowish-brown (Figure S5), which made the recognition of γ-GGT enzyme, was through the naked eyes. In the fluorescent spectra, obvious NIR signal emission was gathered (Figure 1B) accompanied by the quantum yield varying from 0.012 to 0.038 in PBS buffer solution. Notably, such a large stokes shift of Δλ = 210 nm effectively avoiding self-absorption is more conducive to fluorescence imaging (Figure 1C). With the increase of γ-GGT concentration (0-120 mU/mL), the fluorescence intensity around 650 nm of probe NIR-SN-GGT (10 μM) gradually enhanced (Figure 1D) and reached a plateau needing 70 mU/mL γ-GGT with a remarkable 21.84-fold enhancement (Figure 1E). In order to explore the sensitivity of probe NIR-SN-GGT, the low concentration (0–10 mU/mL) titration experiments were carried out in PBS buffer solution (0.01 M, pH 7.4). As is depicted in Figure 1F, there was an excellent linear relationship (*R*^2^ = 0.9851) between the fluorescence intensity of 650 nm and various concentrations. Subsequently, the detection limit (DL) of probe NIR-SN-GGT versus γ-GGT was calculated to be 0.024 mU/mL, and proved that probe had the ability to quantitatively detect trace γ-GGT *in vitro*.

### Response speed and selectivity

**Figure 2:**
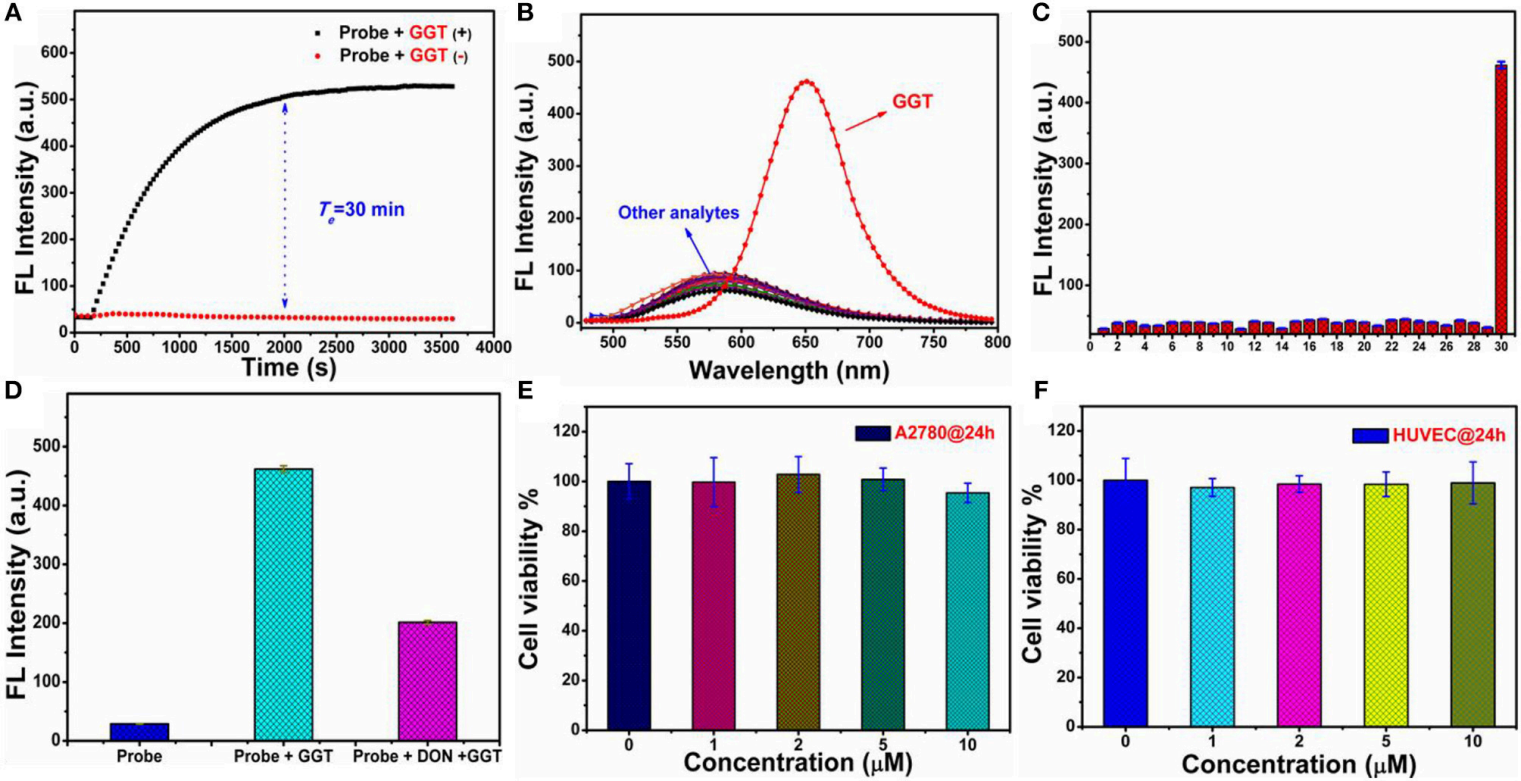
**(A)** Time responses on the fluorescence intensity (F_650nm_) of probe NIR-SN-GGT (10 μM) in the absence (red) and presence (blank) of 100 mU/mL γ-GGT enzyme. **(B)** Fluorescent spectra and **(C)** fluorescent intensity (F_650nm_) changes of probe NIR-SN-GGT (10 μM) for different analytes in PBS buffer solution (0.01 M, pH 7.4). Insert 1: balnk; 2: Na^+^ (500 μM); 3: K^+^ (500 μM); 4: Ca^2+^ (500 μM); 5: Ni^2+^ (500 μM); 6: Mg^2+^ (500 μM); 7: ${\text{NH}}_{4}^{+}$ (500 μM); 8: F^−^ (500 μM); 9: Cl^−^ (500 μM); 10: Br^−^ (500 μM); 11: I^−^ (500 μM); 12: CH_3_COO^−^ (500 μM); 13: ${\text{HCO}}_{3}^{-}$ (500 μM); 14: ${\text{CO}}_{3}^{2-}$ (500 μM); 15: S^2−^ (500 μM); 16: ${\text{HPO}}_{4}^{-}$ (500 μM); 17: ${\text{NO}}_{3}^{-}$ (500 μM); 18: ${\text{SO}}_{4}^{2-}$ (500 μM); 19: SCN^−^ (500 μM); 20: ${\text{NO}}_{2}^{-}$ (500 μM); 21: Glutathione (GSH, 500 μM); 22: Cysteine (Cys, 500 μM); 23: Homocysteine (Hcy, 500 μM); 24: Ascorbic acid (AA, 500 μM); 25: NO (500 μM); 26: NaClO (100 μM); 27: H_2_O_2_ (100 μM); 28: nitroreductase (10 μg/mL); 29 transglutaminase (60 mU/mL); 30: γ-GGT (60 mU/mL). **(D)** Inhibition experiments of probe NIR-SN-GGT (10 μM) for γ-GGT enzyme. **(E,F)** The cytotoxicity of probe NIR-SN-GGT in living A2780 cells and HUVEC cells, respectively. The experiments were repeated three times (cytotoxicity tests for six times) and the data were shown as mean (±S.D.). λ_ex_ = 445 nm, slit: 10/10 nm.

The reaction speed of probe NIR-SN-GGT toward γ-GGT is an important indicator for evaluating the availability of probe. As is seen in Figure 2A, it took approximately 30 min to reach equilibrium which showed that probe NIR-SN-GGT possessed the ability to recognize γ-GGT activity in real-time. Next, biological interferences including ions, amino acid, reductive species, reactive oxygen and enzymes were also investigated the influence on probe. To our delight, except for γ-GGT, none of the others interfered with probe NIR-SN-GGT (Figures 2B,C); clearly indicating that probe NIR-SN-GGT has outstanding selectivity in a variety of biologically related species.

### Effects on micro-environment and sensing mechanism

The influences of micro-environment factors (e.g., pH and temperature) on the recognition of probe NIR-SN-GGT toward γ-GGT were performed. As is demonstrated in Figures S6, S7, the related-parameters (pH = 7.4 and *T* = 37°C) are especially suitable for probe NIR-SN-GGT to track the activity of γ-GGT *in vivo*. Subsequently, we explored the enzyme-activation mechanism of probe for γ-GGT through HPLC and ESI-HRMS experiments. Only probe showed an obvious signal peak with retention time at 13.426 min (Figure S8, blue line). Upon addition γ-GGT into solution for incubation 30 min, a new distinct signal peak appeared at 18.018 min (Figure S8, red line), and the corresponding ESI-HRMS spectra was shown in Figure S9. There was an obvious peak at m/z 290.1658 corresponding to the positive ion mode of product NIR-SN-NH_2_ (calcd. 290.1652 for [M+H]^+^). Besides, the ability catalytic of γ-GGT for probe was largely suppressed (Figure 2D and Figure S10) after adding 30 μM 6-Diazo-5-oxo-L-norleucine (DON, an inhibitor for γ-GGT enzyme). Based on above results, γ-GGT-intervened enzymatic reactions at specific site of probe NIR-SN-GGT was verified shown in Scheme 2.

### MTT assays and cell imaging

In order to study the biocompatibility of probe NIR-SN-GGT, standard 5-diphenyltetrazolium bromide (MTT) assays in A2780 cells and HUVEC cells were carried out, respectively. As is observed in Figures 2E,F, experimental results indicated that probe NIR-SN-GGT was almost no cytotoxicity toward living cells.

**Figure 3:**
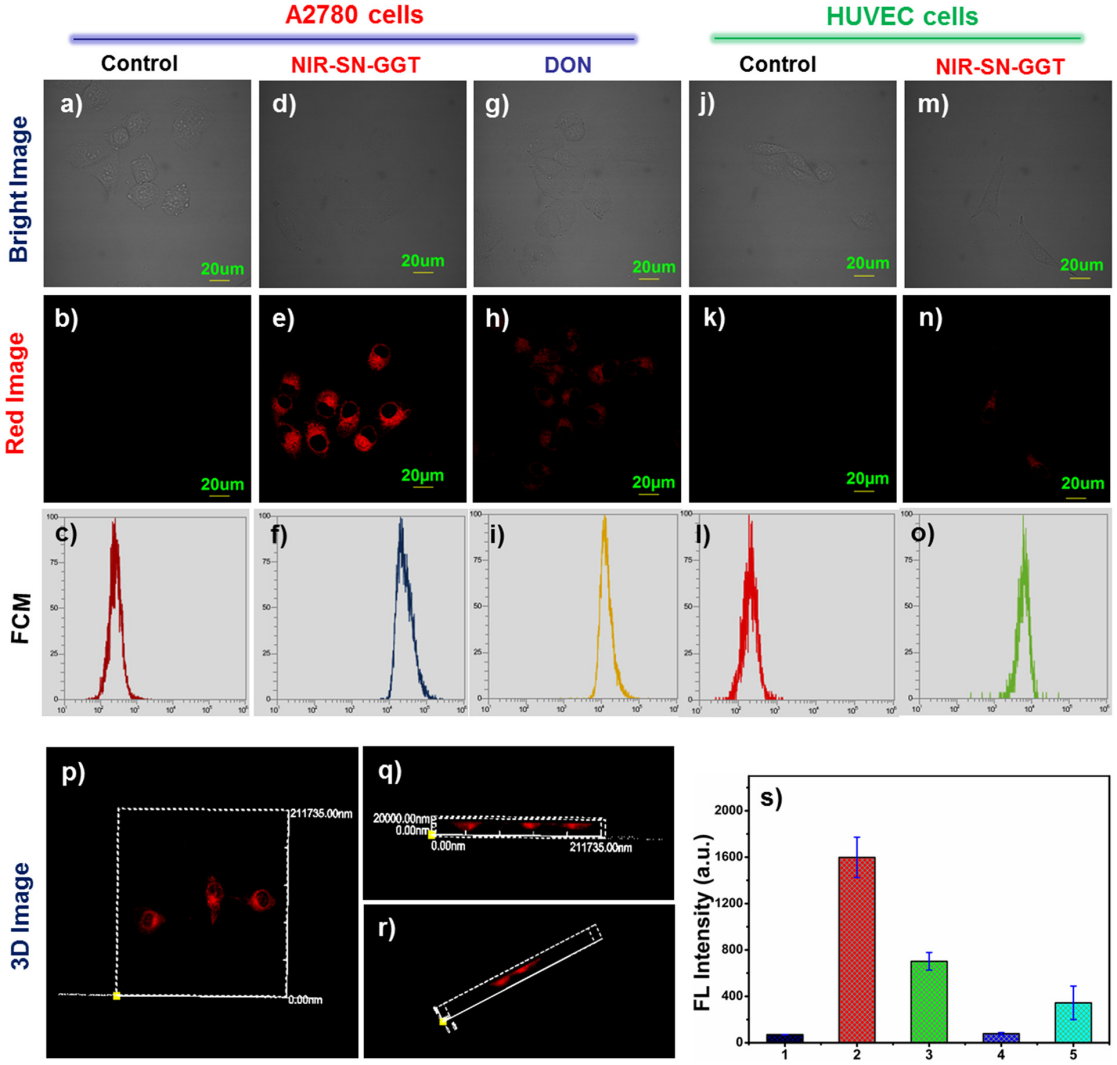
Confocal fluorescence imaging of living cells. **(a,b)** A2780 cells; **(d,e)** A2780 cells incubated with 5 μM probe NIR-SN-GGT; **(g,h)** A2780 cells pretreated with 30 μM DON (inhibitor) and adding 5 μM probe NIR-SN-GGT; **(j,k)** HUVEC cells; **(m-n)** HUVEC cells treated with 5 μM probe NIR-SN-GGT; **(c,f,i,l,o)** represent the corresponding flow cytometry (FCM); **(p,q,r)** 3D fluorescence imaging of A2780 cells; **(s)** fluorescence emission intensities of red channel were measured as averages of 9 regions of interest (ROIs) from different treated cells (**b,e,h,k,n**). Error bar = RSD (*n* = 9). λex = 488 nm and λem = 655–755 nm. Scale bar = 20 μm.

Ovarian cancer cells (cancer cells) were selected as research object because of γ-GGT overexpression on the surface of its membrane. After A2780 cells incubated with 5 μM probe NIR-SN-GGT for 30 min, NIR fluorescence signal (655–755 nm) was obtained from Figure 3e. By contrast, no-treated A2780 cells were basically no fluorescence signal (Figures 3a–c). In addition, pre-treated A2780 cells with 30 μM DON (inhibitor) for 1 h, and then incubated with 5 μM probe NIR-SN-GGT for 30 min, we found that the intensity of NIR fluorescence signal became very weaker (Figures 3g–i) compared to Figures 3d,e. Hence, based on the above experiments confirmed that probe NIR-SN-GGT was used to monitor the activity of γ-GGT through NIR channel. Moreover, the ability of probe for differentiating tumor cells from normal cells was further confirmed. HUVEC cells (normal cells, Figures 3j–l) were used as negative control owing to low expression of γ-GGT. Upon addition 5 μM probe NIR-SN-GGT, negligible fluorescence signal was observed, as expected in Figures 3m,n. Subsequently, flow cytometry assays (FCM) demonstrated that HUVEC cells (Figure 3o) were distinguishable from A2780 cells (Figure 3f) through high throughput data analysis. So, this probe was successfully employed to track γ-GGT enzyme for the identification cancer cells. Besides, three-dimensional (3D) imaging of A2780 cells and HUVEC cells treated with probe NIR-SN-GGT was also investigated *via z*-scan (Figures 3p–r, and Figures S11a–c). Fluorescence emission intensities of NIR channel were measured as averages of 9 regions of interest (ROIs) from different treated cells (Figure 3s). Above results undoubtedly verified that probe NIR-SN-GGT possessed the ability for distinguishing cancer cells from normal cells through 2D&3D imaging, which was beneficial for clinician to the early diagnosis and resection operation of the tumor.

### Time/concentration-dependent imaging

**Figure 4:**
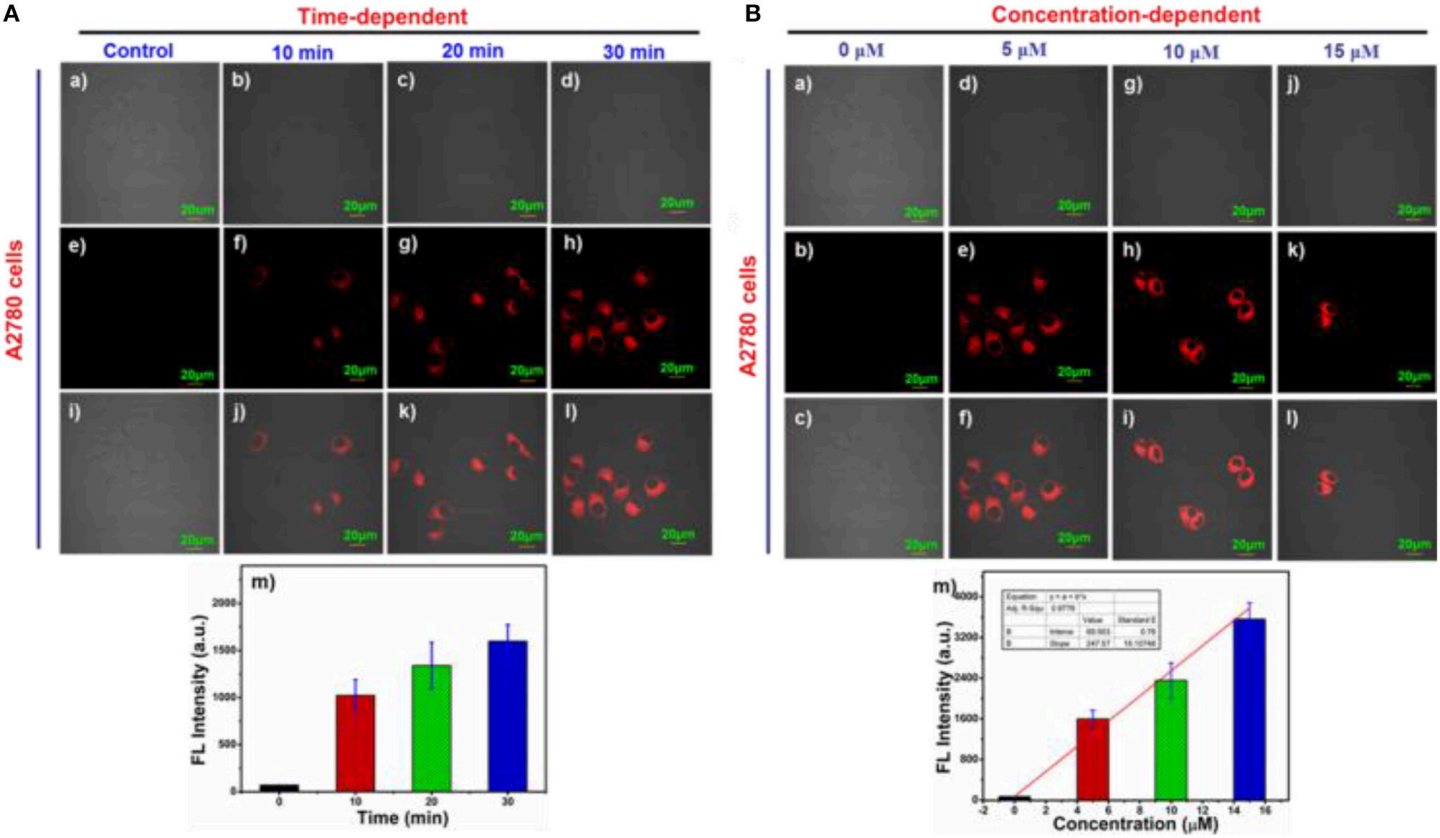
**(A)** Time and **(B)** Concentration-dependent imaging of probe NIR-SN-GGT in A2780 cells. (a–d) bright imaging; (e–h) fluorescence imaging; (i–l) merged imaging; (m) fluorescence emission intensities of NIR channel were measured as averages of 9 regions of interest (ROIs) from different treated A2780 cells. Error bar = RSD (*n* = 9). λex = 488 nm and λem = 655–755 nm. Scale bar = 20 μm.

Time-dependent response of 5 μM probe NIR-SN-GGT in living A2780 cells was carried out. As is seen in Figure 4A, after incubating for 10 min with this probe, there was obvious fluorescence signal from Figures 4Af. As time went on, the fluorescence intensity of NIR channel gradually increased (Figures 4Ae–h,m). By comparison, weak fluorescence signal of HUVEC cells pretreated with 5 μM probe NIR-SN-GGT for 30 min was observed (Figures S12a–i). In addition, concentration-dependent experiments of ovarian cancer cells were also investigated (Figure 4B). With the increase of probe concentration, the fluorescence intensity increased (Figures 4Bb–k) and showed a good linear relationship (*R*^2^ = 0.9776, Figure 4Bm), which verified that probe NIR-SN-GGT could be used to detect the endogenous γ-GGT activity with semi-quantitative detection.

### Two-photon imaging evaluation

**Figure 5:**
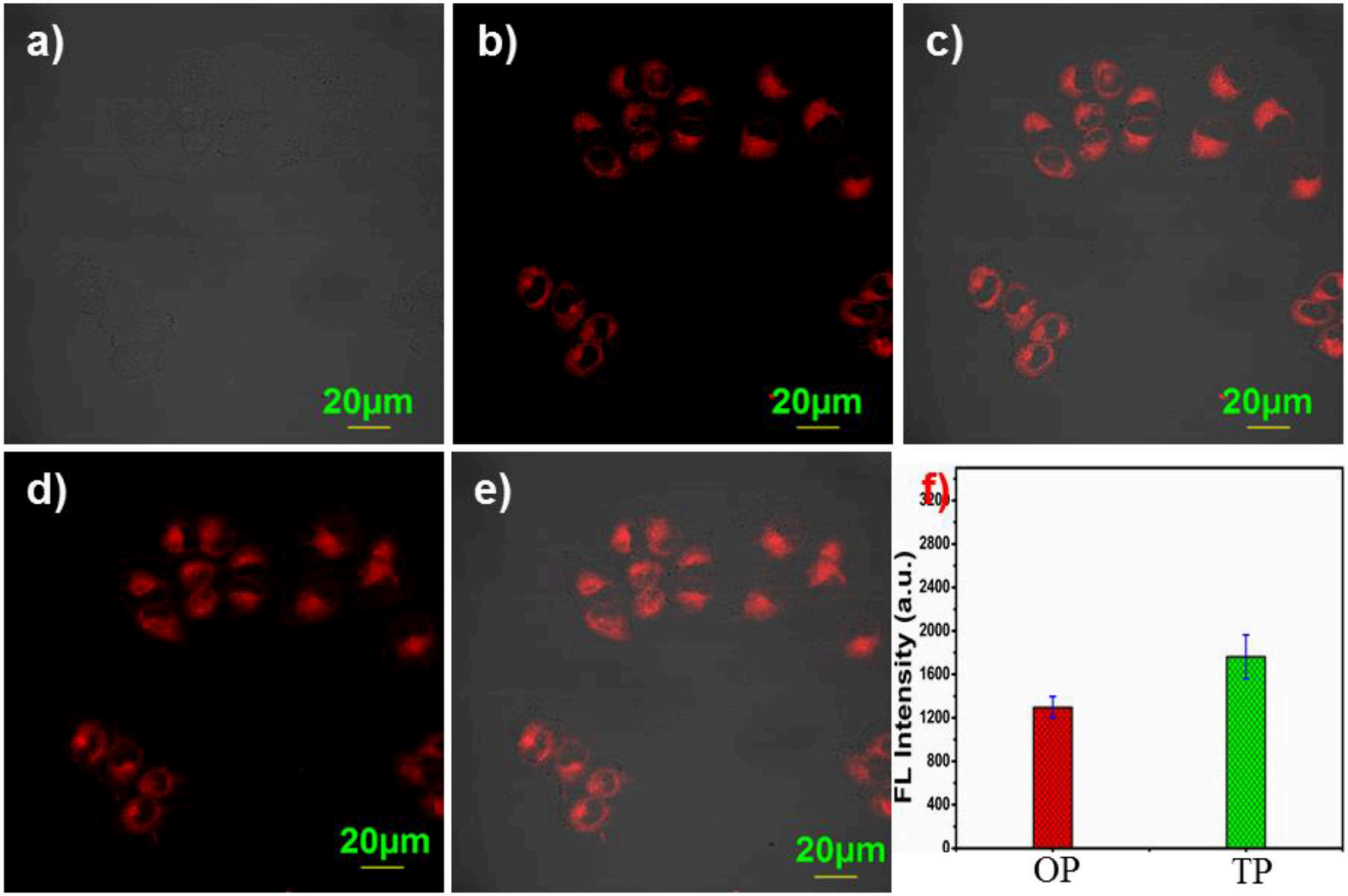
Two-photon fluorescence imaging of probe NIR-SN-GGT in A2780 cells. **(a)** bright imaging; **(b)** one-photon imaging; **(c)** merged imaging from **(a,b)**; **(d)** two-photon imaging; **(e)** merged imaging between **(a)** and **(d)**; **(f)** fluorescence emission intensities of NIR channel were measured as averages of 9 regions of interest (ROIs) from different treated A2780 cells. Error bar = RSD (*n* = 9). (OP mode) λex = 488 nm and λem = 655-755 nm; (TP mode) λex = 800 nm and λem = 575–630 nm. Scale bar = 20 μm.

Considering the advantages of two-photon fluorescence probes, such as long excitation wavelength, deep tissue penetration, and less photo-bleaching etc., are widely used in the field of chemical biology (Li et al., 2017a). Therefore, two-photon (TP) imaging experiments were performed in living A2780 cells. Upon addition 5 μM probe NIR-SN-GGT, obvious two-photon fluorescence signal was obtained (Figures 5d,e) as well as one-photon (OP) imaging (Figures 5a–c,f), indicating that probe NIR-SN-GGT had a two-photon recognition capability for γ-GGT enzyme. Photo-stability is one of the important factors in biological imaging. To our satisfaction, the NIR fluorescence intensity of probe was not apparent changes under continuous two-photon of 800 nm irradiation (Figure S13). Taken together, probe NIR-SN-GGT is an excellent two-photon tool for monitoring the activity of γ-GGT enzyme in living cells with outstanding photo-stability, as we expected.

### Subcellular localization

**Figure 6:**
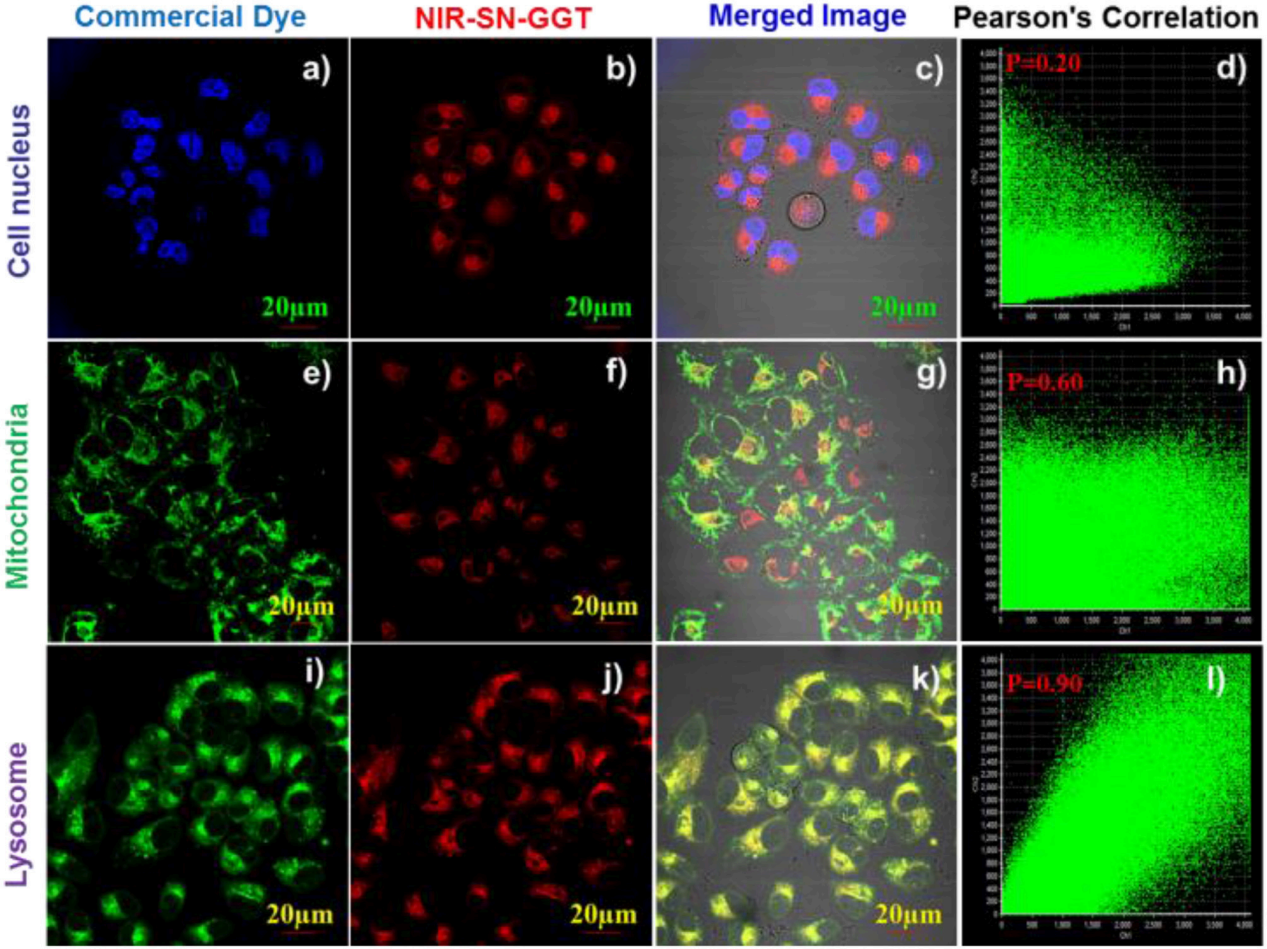
Fluorescence imaging of subcellular localization in living A2780 cells. A2780 cells were treated with 5 μM probe NIR-SN-GGT for 20 min and the co-incubated 0.1 μM Hoechst 33342 **(a–d)**, 0.5 μM Mito-Tracker **(e–h)**, 0.5 μM Lyso-Tracker **(i–l)** for another 15 min at 37 °C. The blue channel of Hoechst 33342 dye was collected at 410-480 nm with the excitation at 405 nm. The green channel of Mito-Tracker and Lyso-Tracker dyes were collected at 500-560 nm with the excitation at 488 nm. The red channel of probe NIR-SN-GGT was collected at 655-755 nm with the excitation at 488 nm. Scale bar = 20 μm.

To research the distribution of probe NIR-SN-GGT triggered by γ-GGT in living A2780 cells, thus, co-location experiments were carried out. As is observed in Figure 6, the red signal of probe NIR-SN-GGT (Figure 6j) and the green signal of Lyso-Tracker (Figure 6i) overlay well (Figure 6k) with the outstanding Pearson's correlation of 0.90 (Figure 6l), which showed that the product of enzyme catalytic probe was mainly clustered in lysosomes rather than nucleus (*P* = 0.20, Figure 6d) and mitochondria (*P* = 0.60, Figure 6h). Faced the above results, we speculated that this distribution was caused by the alkalization effect of the exposed amino products (Scheme 2).

### Imaging of endogenous γ-GGT activity in various organs

**Figure 7:**
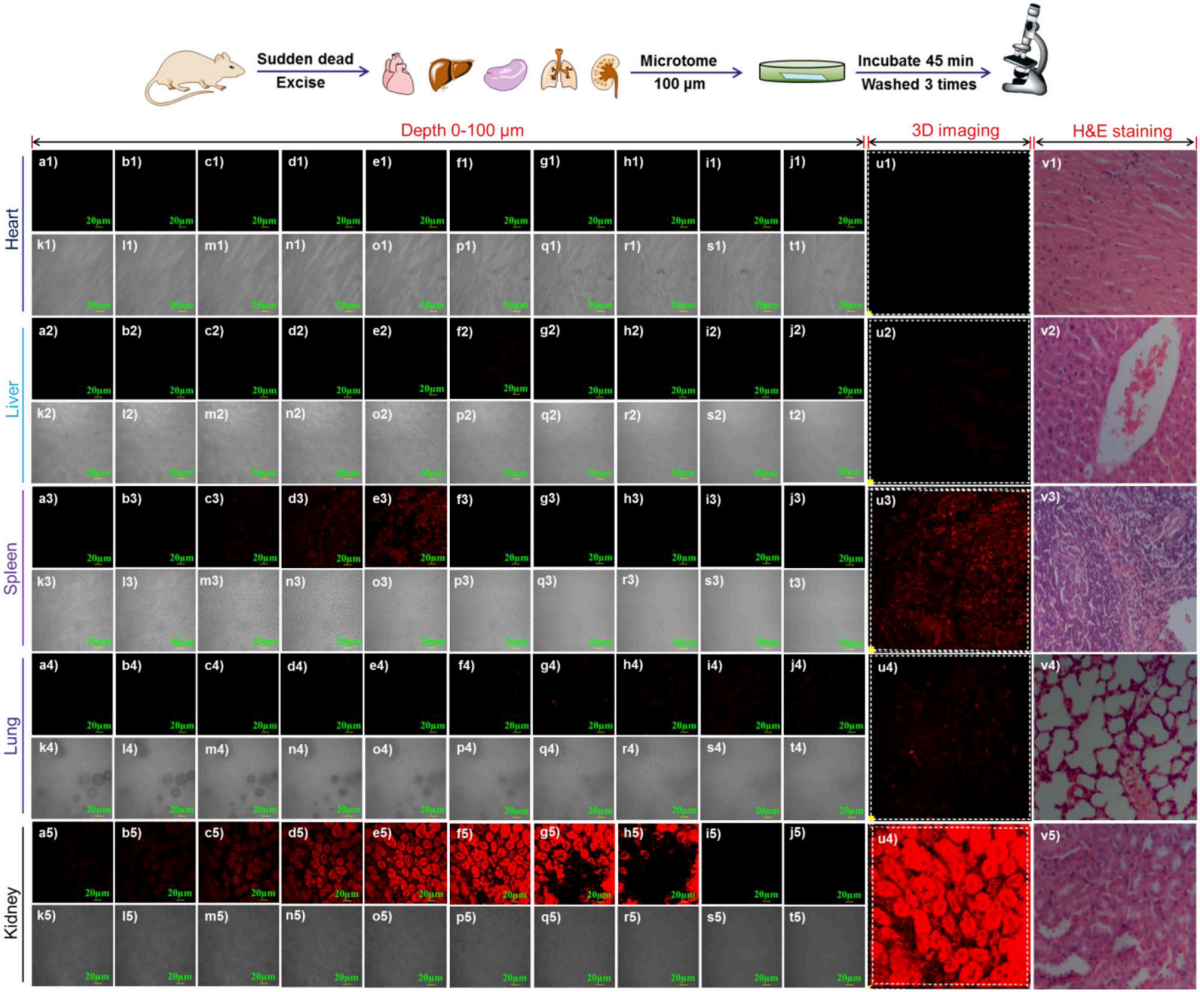
Confocal fluorescence imaging of endogenous γ-GGT activity in various organs including heart, liver, spleen, lung and kidney. Tissue slices of 100 μm were prepared by freezing microtome (LEICA CM1860 UV). 3D-depth images of different tissue were obtained through *z*-scan pattern with step size 10 μm. (a-j) fluorescence channel; k-t bright channel; u) 3D-restruction imaging; v) HandE staining. λex = 488 nm and λem = 655-755 nm. Scale bar = 20 μm.

Endogenous γ-GGT enzyme (Crystal structure, Figure S14) generated from the precursor protein *via* posttranslational processing, catalyzing the cleavage of the γ-glutamyl unit in the important biological species (Okada et al., 2006). Thus, it indicated that γ-GGT plays a very vital role in different tissues and organs. Until now, to the best of our knowledge, there is no literature report to use visualization tool for monitoring the activity of γ-GGT enzyme in various organs. Based on it, we had tried to investigate the applicability of probe NIR-SN-GGT for meeting the above requirements. Tissue sections of the experiment were immediately prepared from isolated organs including heart (Figures 7a1–u1), liver (Figures 7a2–u2), spleen (Figures 7a3–u3), lung (Figures 7a4–u4), and kidney (Figures 7a5–u5) through freezing microtome (LEICA CM1860 UV), and the above organizations biopsy were confirmed through H&E staining (Figures 7v1–v5). Each tissue of 100 μm was pre-treated with 10 μM probe NIR-SN-GGT for 45 min at indoor environment, and washed thrice by PBS buffer to removing excess probe. As shown in Figure 7, the confocal fluorescence imaging of step size 10 μm of tissue slice was performed. Apparent fluorescence signal of nephridial tissue was observed (Figures 7a5–t5), and 3D-reconstructed imaging was shown in Figure 7u5, which clearly visual displayed the content of γ-GGT enzyme more than other organs (heart, liver, spleen, and lung). According to medical research reported glomerulonephritis could increase the γ-GGT enzyme level (Malyszko, 2010). Thus, probe NIR-SN-GGT can be employed for helping secretory doctor to diagnose acute glomerulonephritis. In addition, the content of γ-GGT enzyme in spleen was also abundant (Figures 7a3–u3). Various fluorescence intensity of tissues represented different the content of γ-GGT *in situ*. As far as we know, this is the first NIR fluorescence probe for visualizing the contents of different organs by monitoring the activity of γ-GGT enzyme, which is useful for the detection of γ-GGT-related diseases.

### *In vivo* fluorescence and MRI imaging of γ-GGT activity in xenograft tumor mode

**Figure 8:**
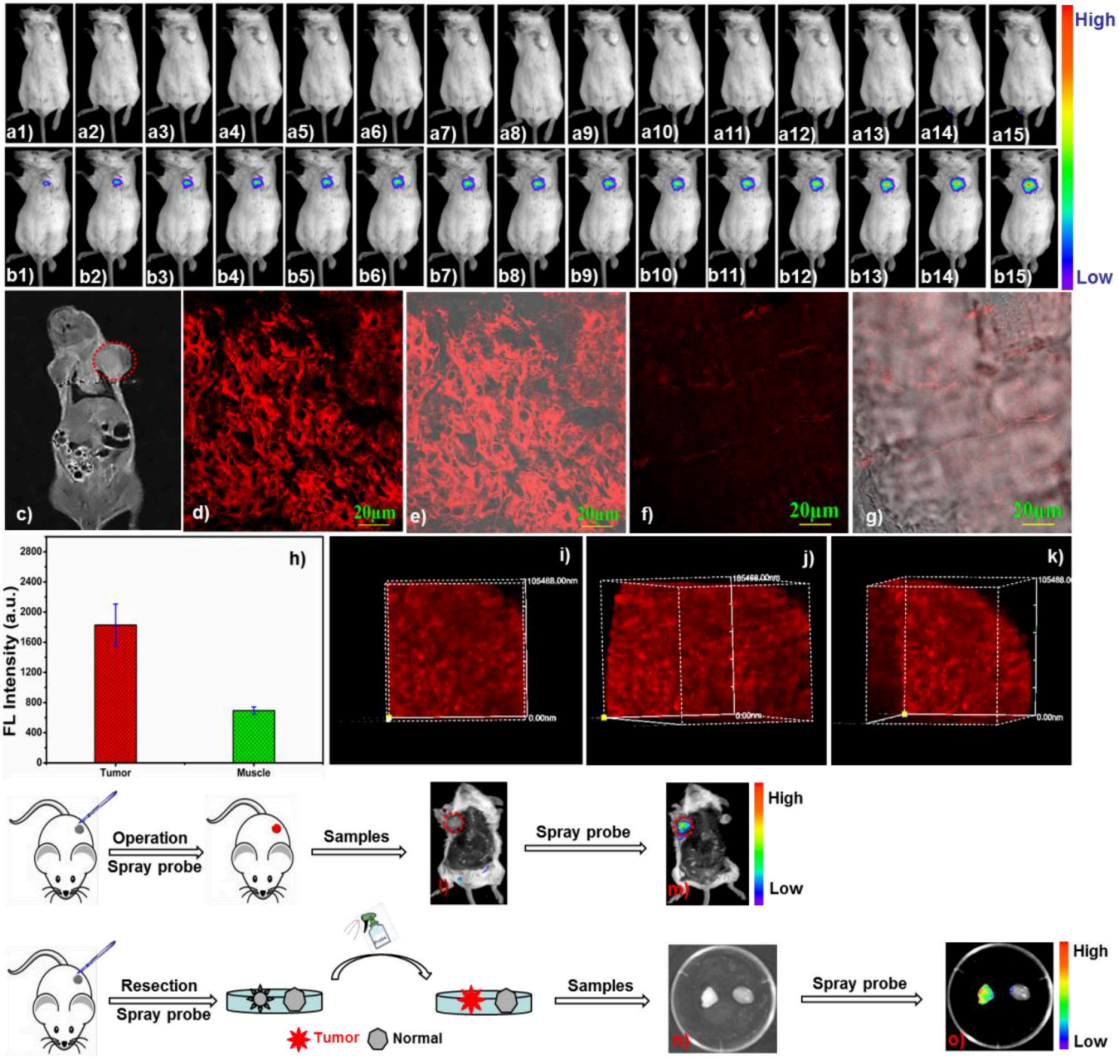
**(a,b)** *In vivo* fluorescence imaging of γ-GGT activity in BABL/c mice bearing xenograft tumor. Probe NIR-SN-GGT (50 μM, 100 μL) was intratumoral injected, subsequently, fluorescent photographs (insert: b1) 2 min; (b2) 5 min; (b3) 10 min; (b4) 15 min; (b5) 20 min; (b6) 25 min; (b7) 30 min; (b8) 40 min; (b9) 50 min; (b10) 60 min; (b11) 70 min; b12 80 min; b13) 90 min; (b14) 100 min; (b15) 120 min) were gathered with excitation at 475 nm (fwhm 20 nm) and emission at 655 nm (fwhm 20 nm). (a1–a15) the corresponding control group was injected equivalent PBS buffer (pH 7.4, 0.01 M) solution. **(c)** MRI imaging (MesoMR23-060H-I) *via* T2 weighted imaging (red dotted line represents the tumor). **(d–g)** Tissue imaging of tumor tissue (**d**-fluorescence channel, **e**-merged channel) and normal (**f**-fluorescence channel, **g**-merged channel) pre-incubated with 10 μM probe NIR-SN-GGT in PBS buffer solution, and washed thirce by PBS buffer. **(h)** The fluorescence intensities of tumor and normal were gathered as averages of 21 regions of interest (ROIs) from NIR channel. **(i–k)** 3D-depth imaging (500 μm) of tumor tissue under the two-photon excitation at 800 nm. Fluorescence imaging of mouse (before **l**) and after **(m)** was sprayed with probe NIR-SN-GGT (100 μM, 150 μL). Fluorescence imaging of tumor tissue (left side) and normal tissue (right side) *via* “spraying” style (**n**: bright image; **o**: fluorescence image). λex = 488 nm, λem = 655–755 nm. Scale bar = 20 μm.

In view of its high selectivity, sensitivity, photo-stability as well as NIR fluorescence emission, avoiding background interference and reducing light scattering, has been laid the foundation for *in vivo* application. Then, we explored the availability of probe NIR-SN-GGT in the transplanted tumor of BABL/c mice. As demonstrated in Figure 8b, after probe NIR-SN-GGT (50 μM, 100 μL) was directly *in-situ* injected, the NIR fluorescence signal of 655-755 nm on the tumor area enhanced gradually (Figures 8b1–b15), which attributed to the tracking over-expression γ-GGT on tumor site. In comparison, fluorescence intensity of control group injected equivalent PBS buffer solution was not gathered up to 2 h (Figures 8a1–a15). Combined with magnetic resonance imaging (MRI) through T2 weighted imaging (Figure 8c), probe NIR-SN-GGT could light up tumor relying on tracking endogenous γ-GGT enzymes, which would help surgeon real-time to the diagnosis *in situ* and treatment of cancer. Besides, tissues imaging of probe NIR-SN-GGT were also performed on laser confocal fluorescence microscopy (FV1000-IX81). Various tissues samples were prepared by freezing-microtome (Leica CM1860 UV), and pretreated with 10 μM probe NIR-SN-GGT for 45 min at room temperature. Obvious NIR fluorescence signal of 655-755 nm was gathered from tumor tissue, as we expected in Figures 8d,e. By comparison, weak fluorescence signal of normal tissue was observed due to lacking of γ-GGT overexpression (Figures 8f,g and Figure S16b). Their fluorescence intensity statistics were shown in Figure 8h, which were consistent with layer scanning of tissue imaging (Figure S15). In consideration of its excellent two-photon performance, 3D imaging of 500 μm was also studied under the excitation 800 nm by *z*-scan pattern with step size 5 μm. As depicted in Figures 8i–k, remarkable 3D-imaging clearly demonstrated probe NIR-SN-GGT had excellent deep tissue permeability. More interestingly, tumor could be lighted up (Figures 8l,m) after “spraying” probe NIR-SN-GGT (100 μM, 150 μL) 30 min, greatly promoting the possibility of precise excision. In addition, compared to normal tissue (Figures 8n,o, right side), isolated tumor tissue (Figure S16a, H&E staining) was also easily lighted up through “spraying” probe NIR-SN-GGT (100 μM, 50 μL) on the surface (Figures 8l,m, left side). Based on above results, to our satisfaction, probe NIR-SN-GGT was used to the identification tumor transplanted in BABL/c mice and deep tissue (up to 500 μm) accompanied by MRI imaging, which would be in favor of early diagnosis and treatment of tumor in clinical practice.

## Conclusion

In summary, a smart fluorescent probe NIR-SN-GGT was successfully developed through rational design. After γ-GGT accurately cleaving activated unit, liberating bare amino, NIR fluorescence emission signal of 650 nm strengthen gradually, which attributed to the recovery of Intramolecular Charge Transfer (ICT) mechanism. Only γ-GGT made the probe fluorescence spectrum clear change, and low detection limit of probe was calculated to be 0.024 mU/mL, undoubtedly verified that probe NIR-SN-GGT would employed to detect trace γ-GGT in complex system. Owing to its good biocompatibility and photo-stability, probe NIR-SN-GGT was also used to the identification cancer cells (A2780 cells) from normal cells (HUVEC cells) by fluorescence imaging and high throughput flow cytometry. Through “spraying” manner, to the best of our knowledge, tumor tissue could be lighted up compared to normal tissue for first time. We hope that enzyme-activated NIR fluorescent probe NIR-SN-GGT can be a potential functional molecular tool for inhibitor development, early diagnosis and resection of cancer.

## Ethics statement

This study was carried out in accordance with the recommendations of Dalian Medical University Animal Care and Use Committee. The protocol was approved by the Dalian Medical University Animal Care and Use Committee.

## Author contributions

HL was responsible for performing the experiments and writing manuscript. QY was responsible for carrying out animal experiments. FX and NX were responsible for providing cells. WS, SL, JD, and JF were responsible for discussing experimental results. JW and XP were responsible for designing experiments and revising the paper.

### Conflict of interest statement

The authors declare that the research was conducted in the absence of any commercial or financial relationships that could be construed as a potential conflict of interest.
