# Supplementary material for: Lighting-Up Tumor for Assisting Resection via Spraying NIR Fluorescent Probe of γ-Glutamyltranspeptidas
Source: Front Chem. 2018 Oct 12;6:485. doi: 10.3389/fchem.2018.00485 (PMC6194167; doi:10.3389/fchem.2018.00485)
Supplement: Supplementary file 1 [file Table_1.DOCX]

Supplementary Material

Lighting-up Tumor for Assisting Resection via Spraying NIR Fluorescent Probe of γ-Glutamyltranspeptidase

Haidong Li^1^, Qichao Yao^1^, Feng Xu^1^, Ning Xu^1^, Wen Sun^1^, Saran Long^1^, Jianjun Du^1^, Jiangli Fan^1^, Jingyun Wang^2^, Xiaojun Peng^1*^

^1^ State Key Laboratory of Fine Chemicals, Dalian University of Technology, 2 Linggong Road, High-tech District, Dalian 116024, China.

^2^ Department School of Life Science and Biotechnology, Dalian University of Technology, 2 Linggong Road, High-tech District, Dalian 116024, China.

*** Correspondence:** Xiaojun Peng: [pengxj@dlut.edu.cn](mailto:pengxj@dlut.edu.cn)

**Contents**

**Figure S1.** UV-Vis spectra of probe NIR-SN-GGT in various solutions…………………...…Page S3

**Figure S2.** Fluorescence spectra of probe NIR-SN-GGT in various solutions……………...…Page S3

**Figure S3.** UV-Vis spectra titration tests of probe NIR-SN-GGT in PBS solution………...….Page S4

**Figure S4.** Stability of probe NIR-SN-GGT (10 μM) in PBS solution………………………...Page S4

**Figure S5.** The color change of the solution of probe NIR-SN-GGT……………………….…Page S5

**Figure S6.** pH effects on probe NIR-SN-GGT (10 μM)………………………………….……Page S5

**Figure S7.** Temperature effects on probe NIR-SN-GGT (10 μM)……………………….……Page S6

**Figure S8.** HPLC assays…………………………………………………………...……….….Page S6

**Figure S9.** ESI-HRMS verify mechanism………………………………………………...…...Page S7

**Figure S10.** Inhibition test of probe NIR-SN-GGT…………………….………………....…...Page S7

**Figure S11.** 3D fluorescence imaging of HUVEC cells……………………………..….……..Page S8

**Figure S12.** Time-dependent imaging of probe NIR-SN-GGT in HUVEC cells………...…....Page S8

**Figure S13** Photo-stability evaluation of probe NIR-SN-GGT in living cells…………………Page S9

**Figure S14** Crystal structure of γ-GGT from *Escherichia coli*…………………………..….…Page S9

**Figure S15** Confocal fluorescence imaging of endogenous γ-GGT activity in muscle tissue and Tumor tissue……………………………………………………………………………...…...Page S10

**Figure S16** H&E staining of cancer and normal tissue………………...….………….………Page S10

**Figure S17-22** ^1^H-NMR, ^13^C-NMR and MS of compounds………………………….………Page S11


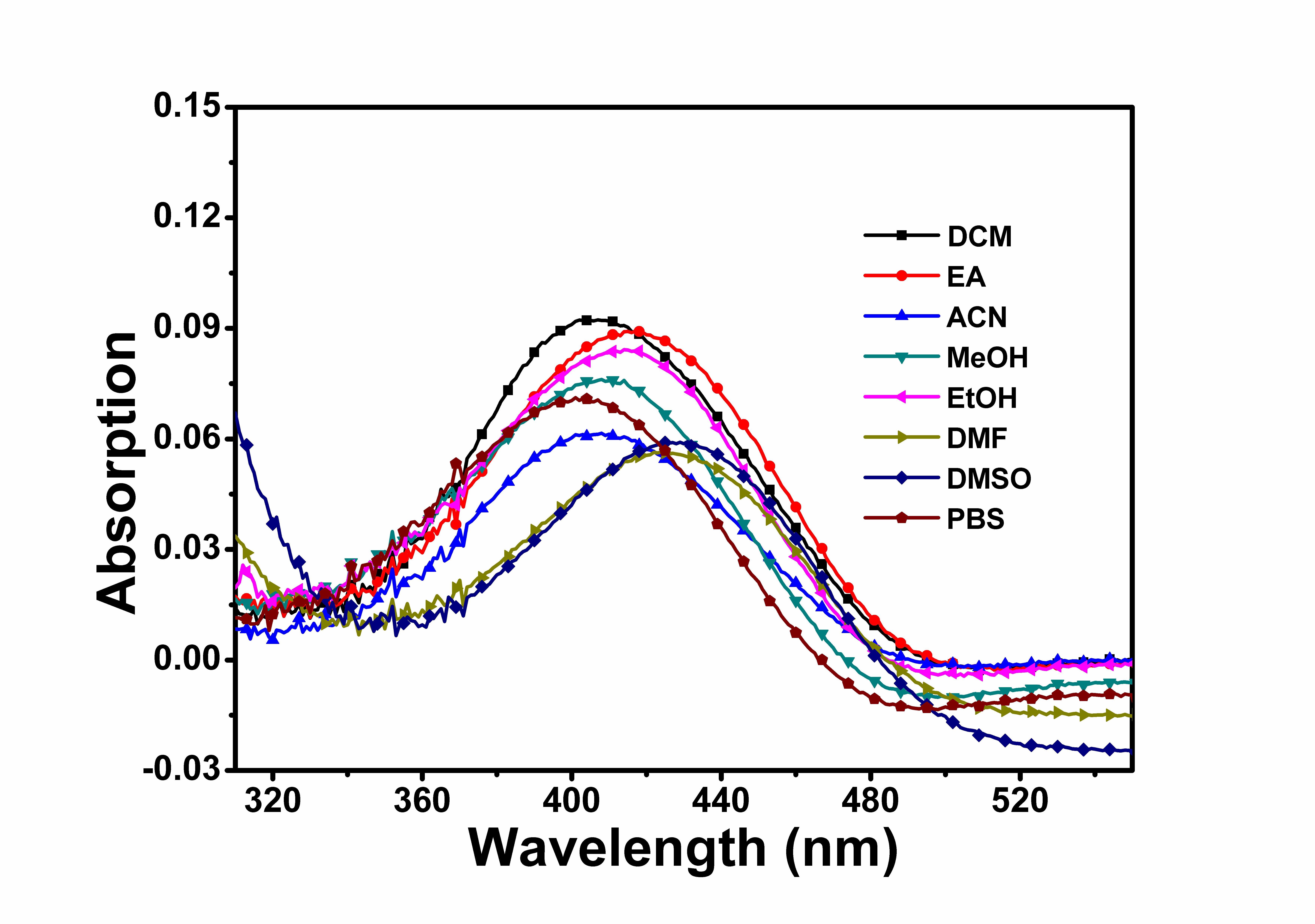


**Figure S1.** UV-Vis spectra of probe NIR-SN-GGT in various solutions.


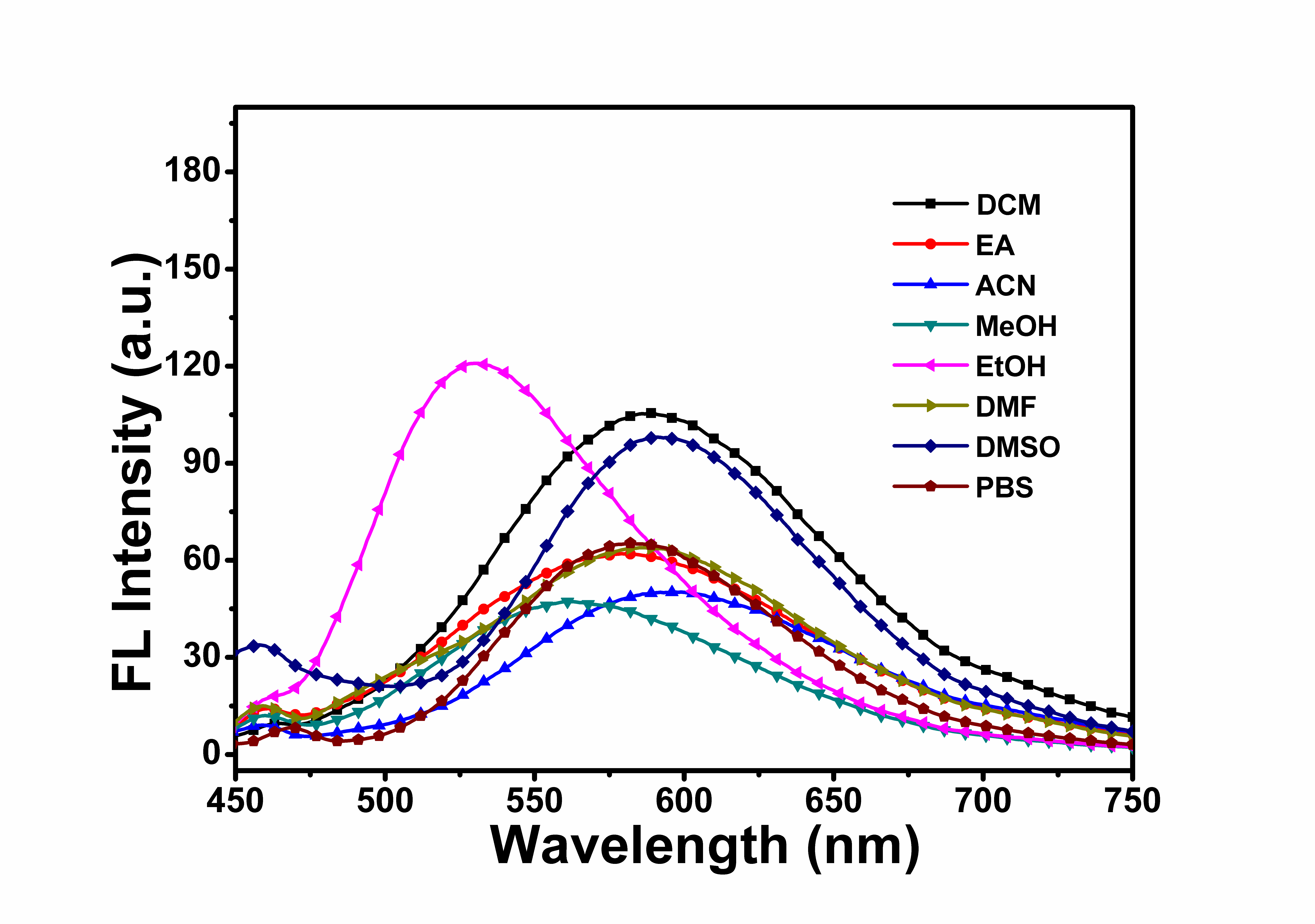


**Figure S2.** Fluorescence spectra of probe NIR-SN-GGT in various solutions.


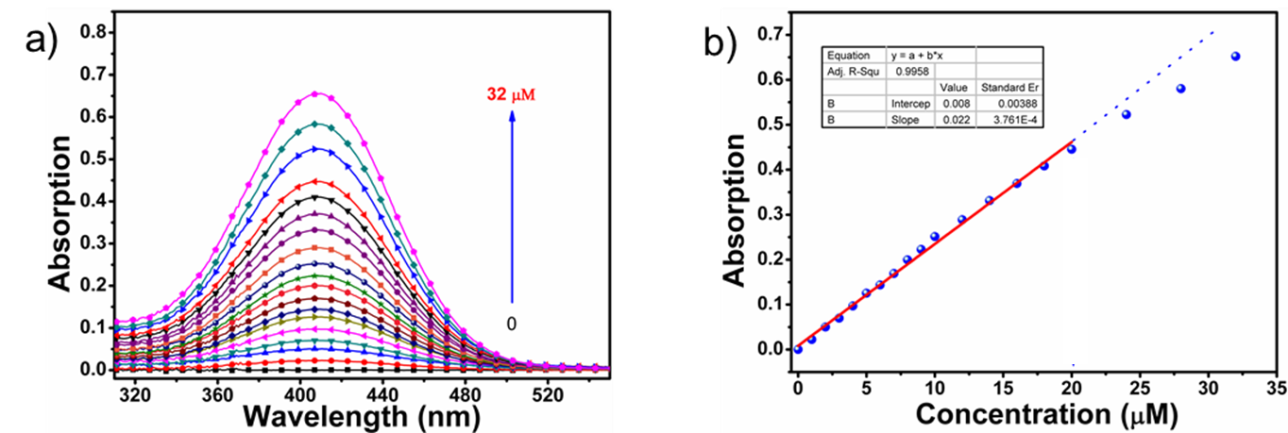


**Figure S3.** UV-Vis spectra titration tests of probe NIR-SN-GGT in PBS solution (0.01 M, pH 7.4).

**
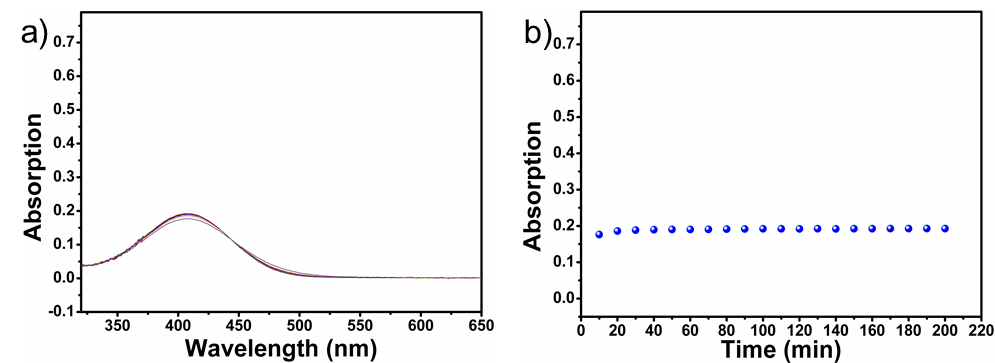
**

**Figure S4.** Stability of probe NIR-SN-GGT (10 μM) in PBS solution (0.01 M, pH 7.4).


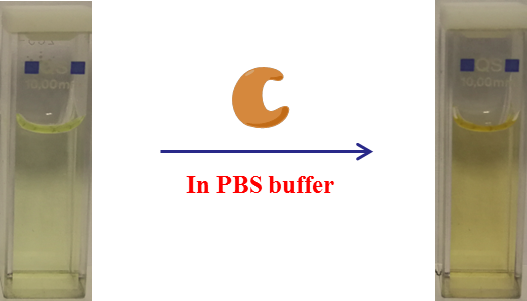


**Figure S5.** The color change of the solution of probe NIR-SN-GGT (10 μM) in present of 60 mU/mL γ-GGT in PBS buffer solution (0.01 M, pH 7.4).


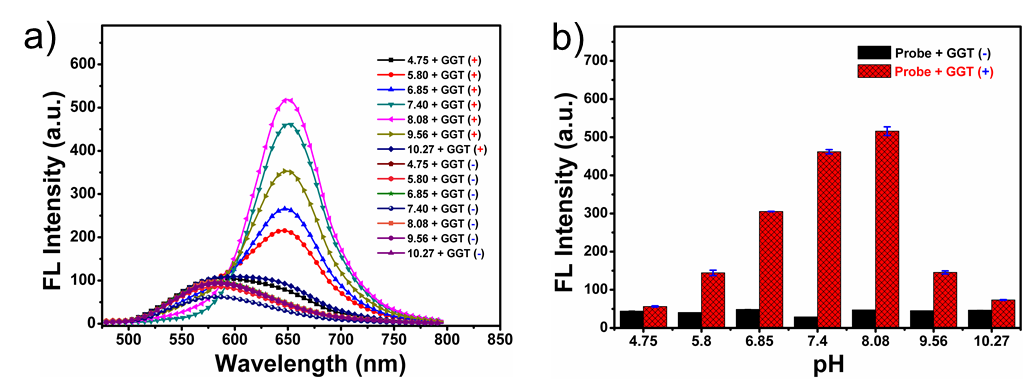


**Figure S6.** pH effects on probe NIR-SN-GGT (10 μM) and its activity toward 60 mU/mL γ-GGT in PBS buffer solution.


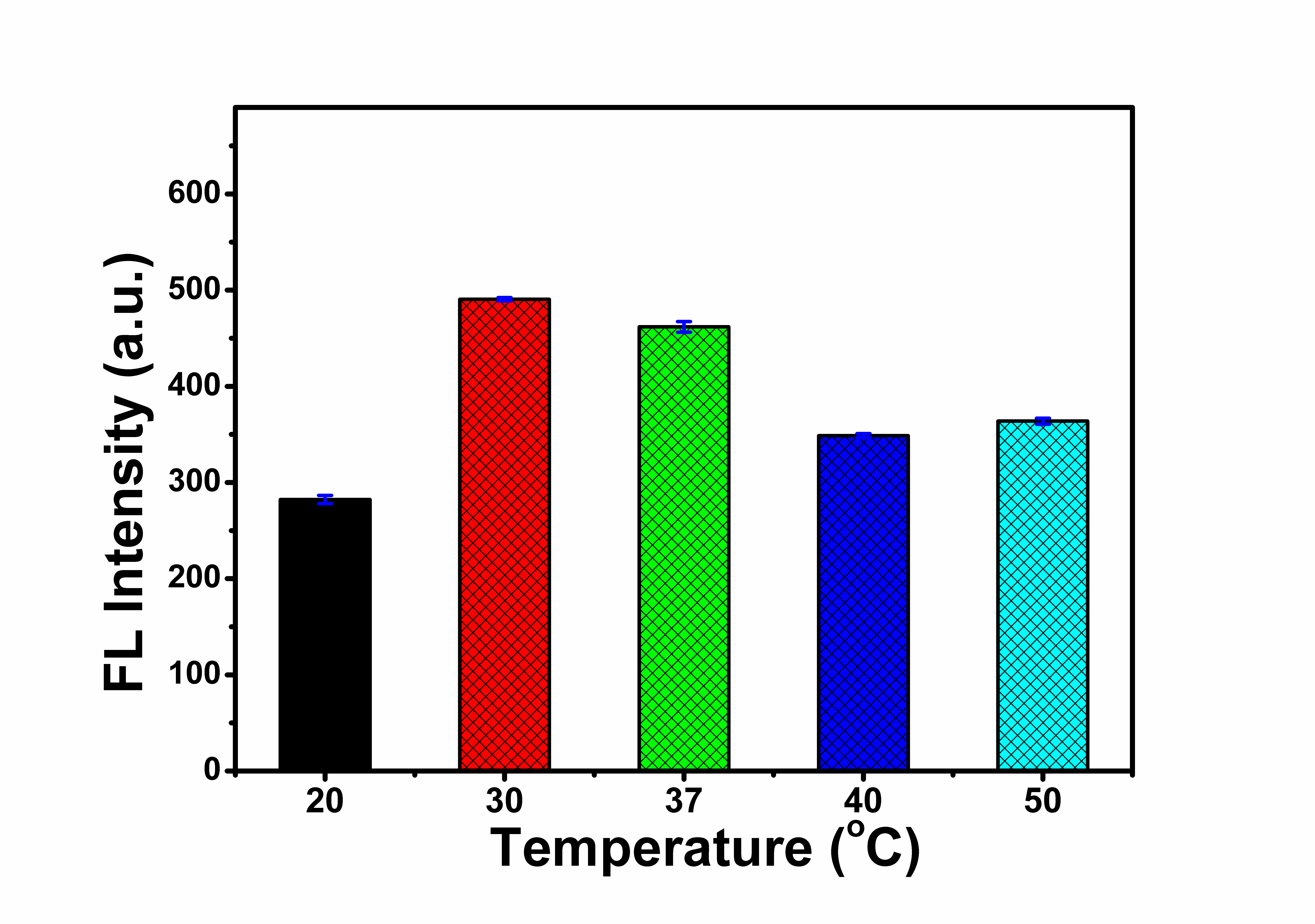


**Figure S7.** Temperature effects on probe NIR-SN-GGT (10 μM) toward 60 mU/mL γ-GGT in PBS buffer solution (0.01 M, pH 7.4).


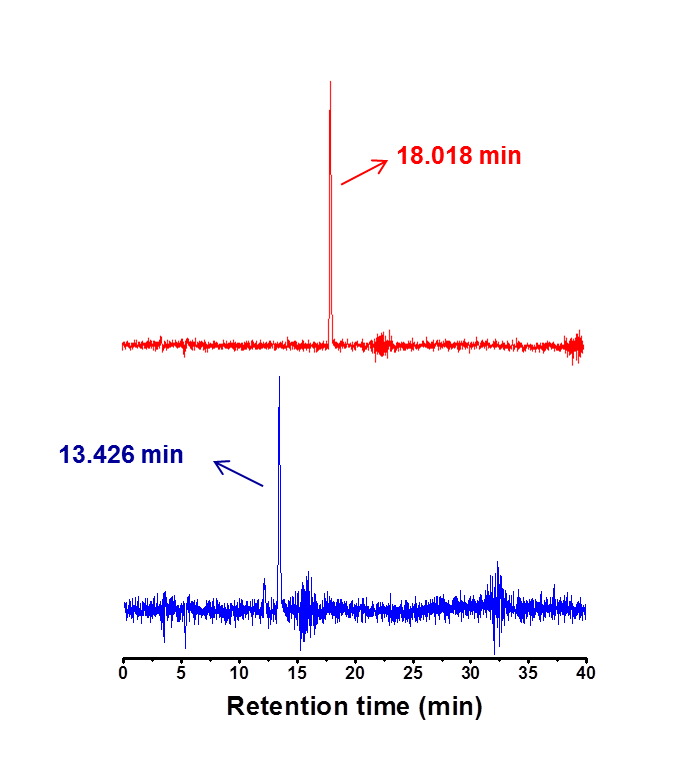


**Figure S8.** HPLC assays of probe NIR-SN-GGT in the absence (blue) and presence (red) of γ-GGT enzyme.


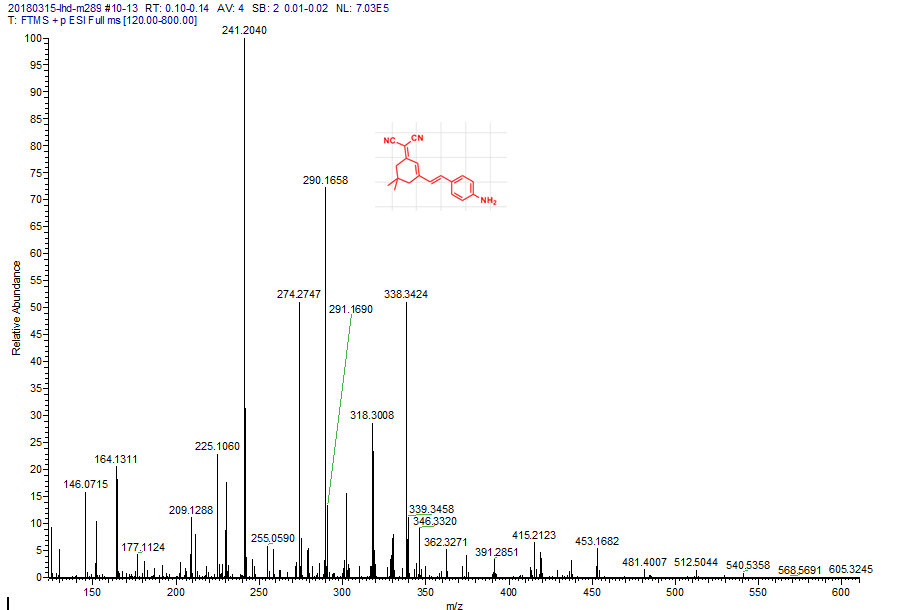


**Figure S9.** ESI-HRMS verify mechanism


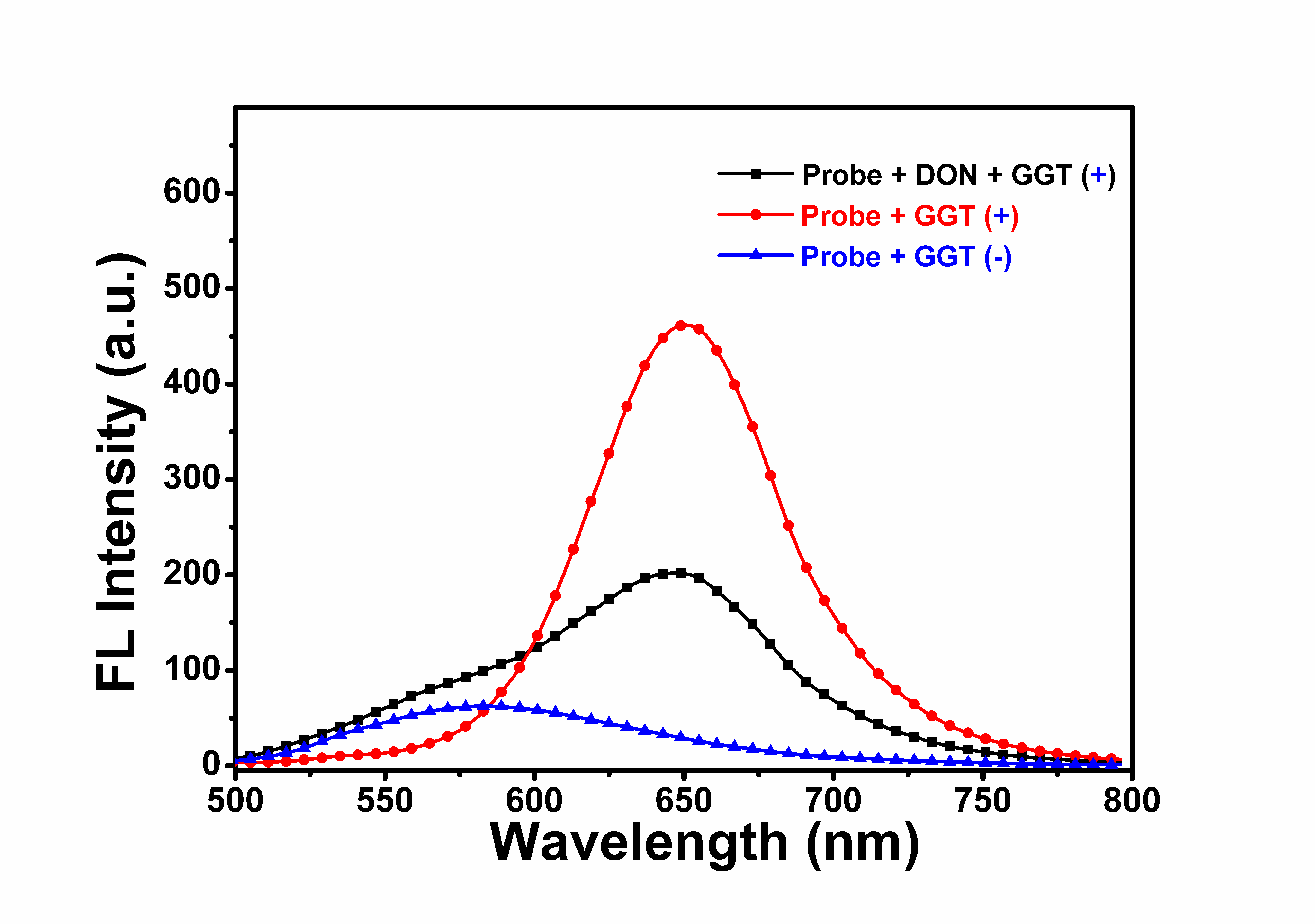


**Figure S10.** Inhibition test of probe NIR-SN-GGT for γ-GGT enzyme in PBS buffer. Insert blue line: Only 10 μM NIR-SN-GGT; red line: 10 μM NIR-SN-GGT + 60 mU/mL γ-GGT; black line: 10 μM NIR-SN-GGT + 60 mU/mL γ-GGT + 30 μM DON.


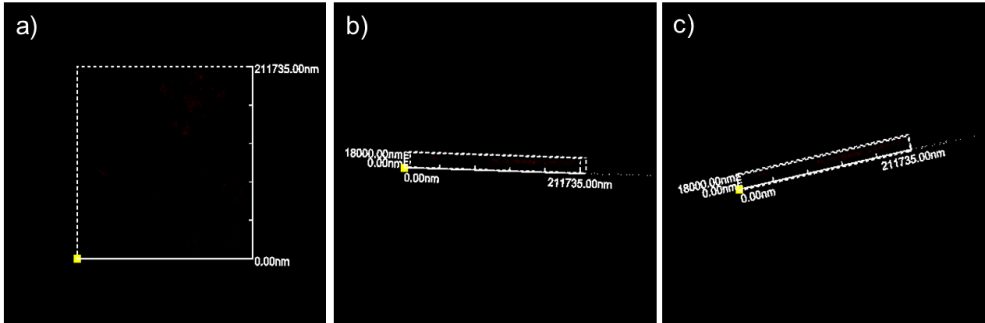


**Figure S11.** 3D fluorescence imaging of HUVEC cells pre-treated with 5 μM NIR-SN-GGT.


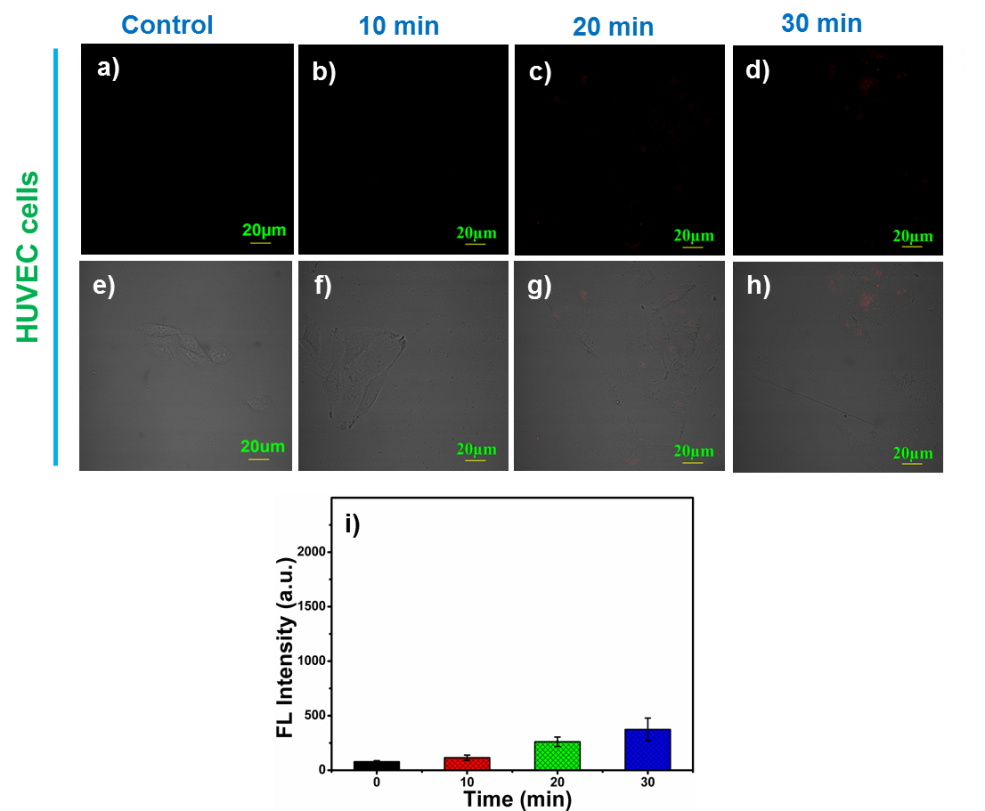


**Figure S12.** Time-dependent imaging of probe NIR-SN-GGT in HUVEC cells. a-d) bright imaging; e-h) merged imaging; i) fluorescence emission intensities of NIR channel were measured as averages of 9 regions of interest (ROIs) from different treated HUVEC cells. Error bar = RSD (n=9). λex = 488 nm and λem = 655-755 nm. Scale bar = 20 μm.


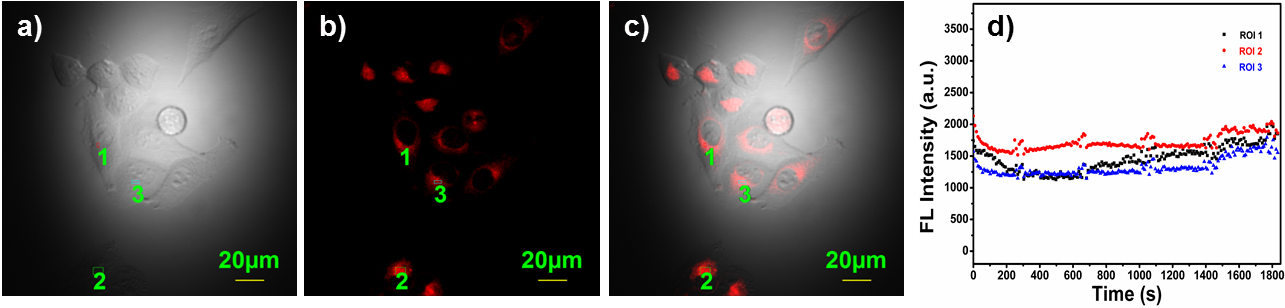


**Figure S13.** Photo-stability evaluation of probe NIR-SN-GGT in living cells through continuous two-photon excitation of 800 nm. Scale bar = 20 μm.


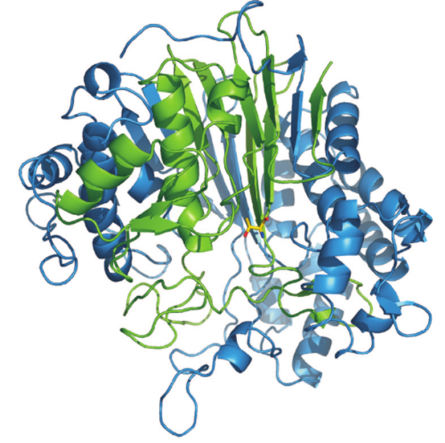


**Figure S14.** Crystal structure of γ-GGT from *Escherichia coli* (Okada. et al., 2006).


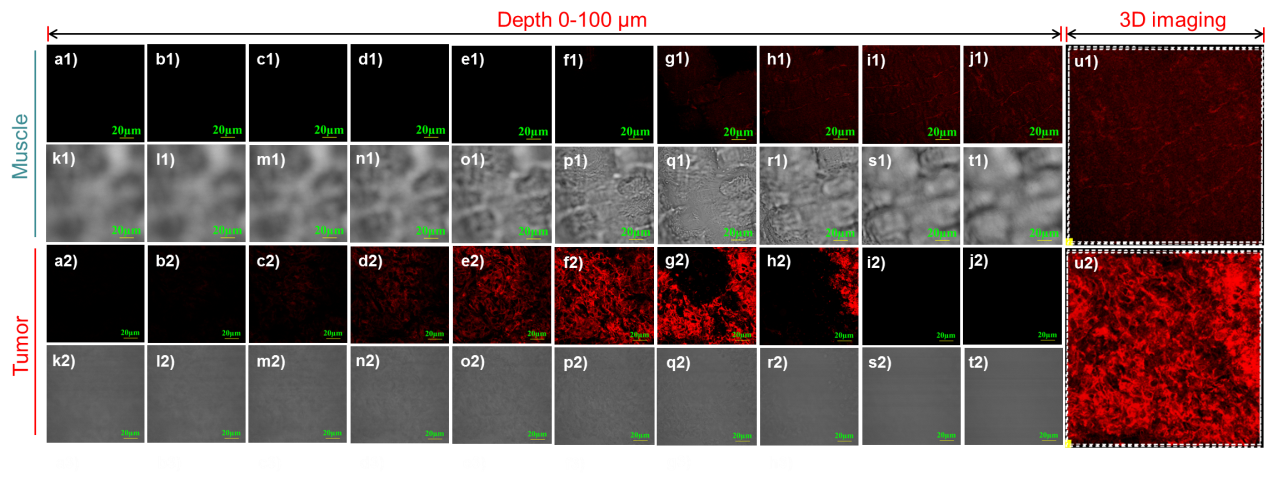


**Figure S15.** Confocal fluorescence imaging of endogenous γ-GGT activity in muscle tissue (a1-u1) and tumor tissue (a2-u2). Tissue slices of 100 μm were prepared by freezing microtome (LEICA CM1860 UV). 3D-depth images of different tissue were obtained through z-scan pattern with step size 10 μm. a-j) fluorescence channel; k-t) bright channel; u) 3D-restruction imaging. λex = 488 nm and λem = 655-755 nm. Scale bar = 20 μm.


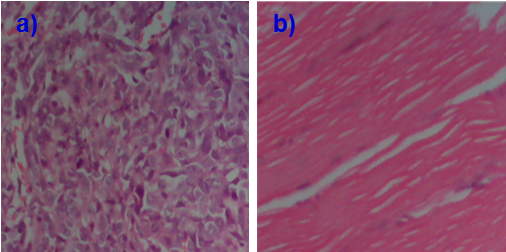


**Figure S16.** H&E staining of a) cancer tissue and b) normal tissue.


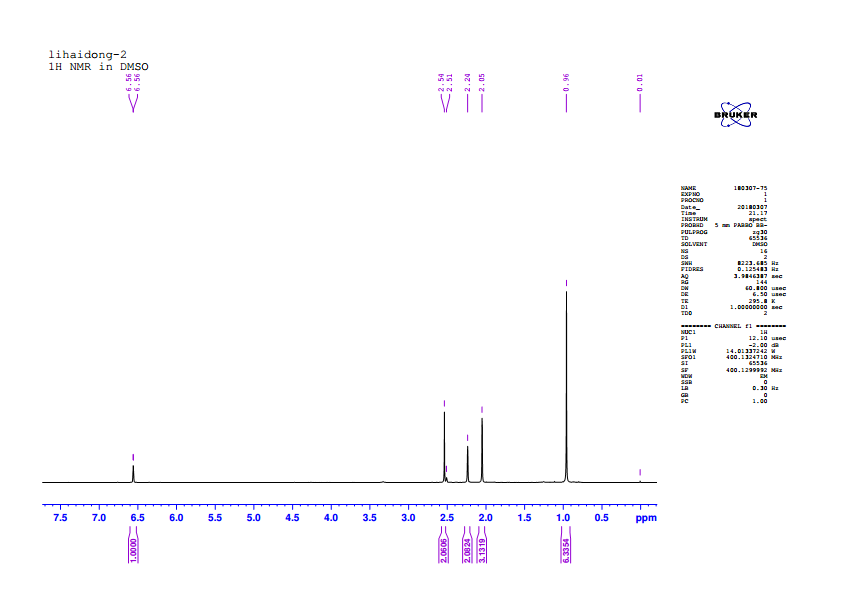


**Figure S17.** ^1^H NMR of compound 2 in DMSO-*d_6_*.


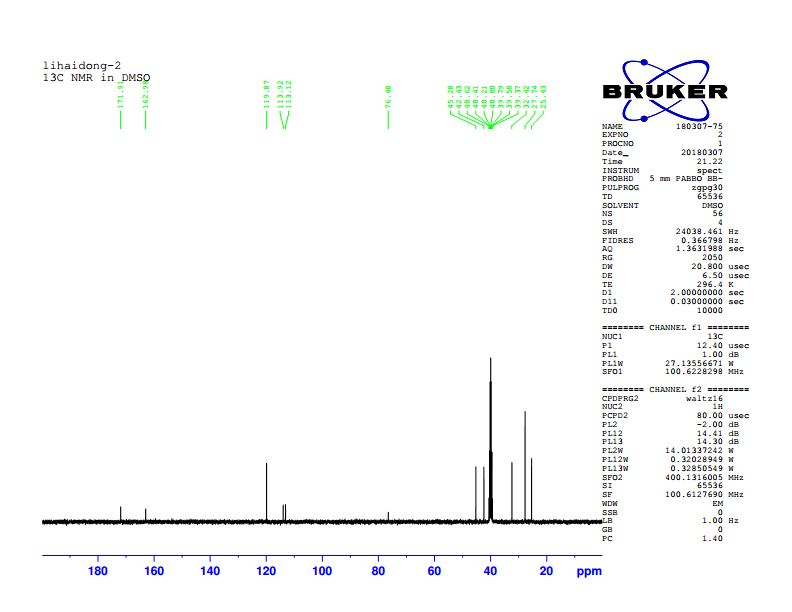


**Figure S18.** ^13^C NMR of compound 2 in DMSO-*d_6_*.

**Figure S19.** MS of compound 2.


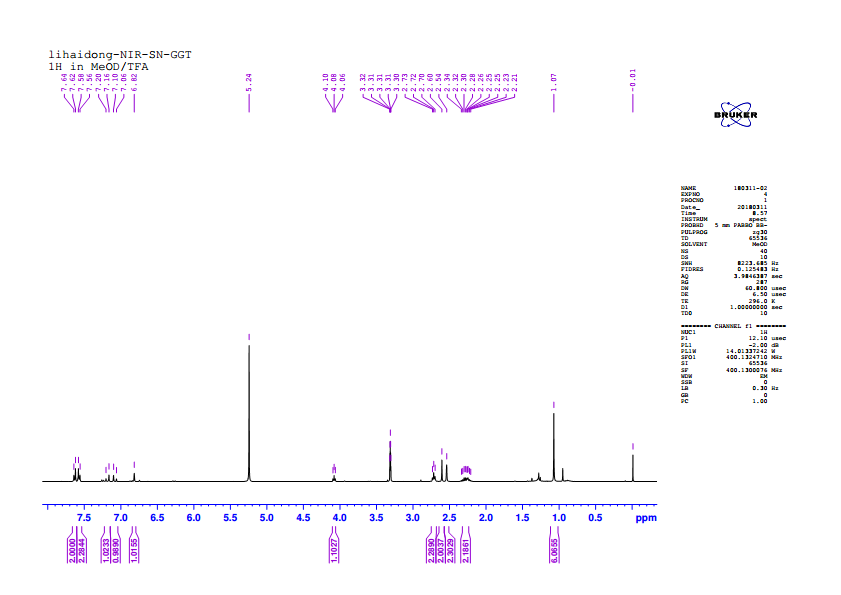


**Figure S20.** ^1^H NMR of probe NIR-SN-GGT in MeOD-TFA.


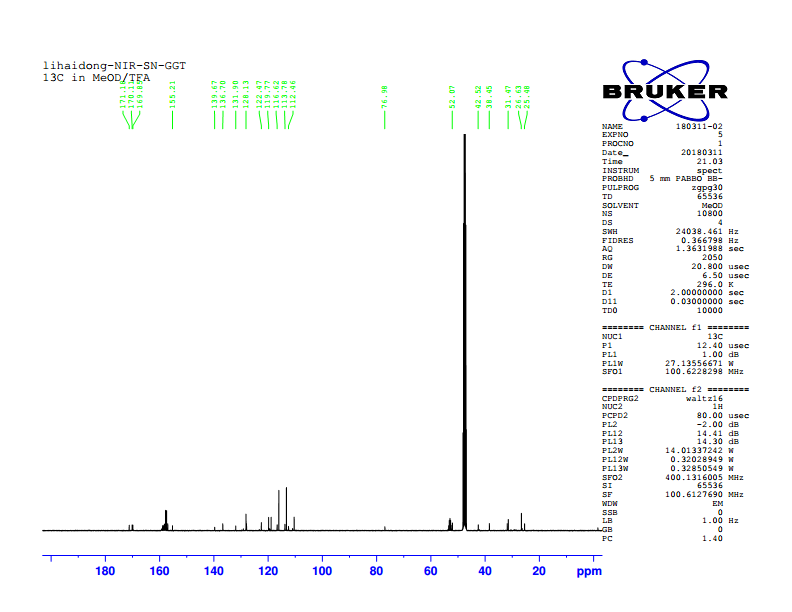


**Figure S21.** ^13^C NMR of probe NIR-SN-GGT in MeOD-TFA.

**Figure S22.** ESI-HRMS of probe **NIR-SN-GGT**.

Okada, T., Suzuki, H., Wada, K., Kumagai, H., and Fukuyama, K. (2006). Crystal structures of gamma-glutamyltranspeptidase from Escherichia coli, a key enzyme in glutathione metabolism, and its reaction intermediate. *P. Natl. Acad. Sci. USA* 103, 6471-6476. doi: 10.1073/pnas.0511020103.
